# Initiation and Flow Conditions of Contemporary Flows in Martian Gullies

**DOI:** 10.1029/2018JE005899

**Published:** 2019-08-28

**Authors:** T. de Haas, B. W. McArdell, S. J. Conway, J. N. McElwaine, M. G. Kleinhans, F. Salese, P. M. Grindrod

**Affiliations:** ^1^ Department of Physical Geography Universiteit Utrecht Utrecht The Netherlands; ^2^ Department of Geography Durham University Durham UK; ^3^ Swiss Federal Institute for Forest, Snow and Landscape Research WSL Birmensdorf Switzerland; ^4^ Laboratoire de Planétologie et Géodynamique, CNRS UMR 6112, Université de Nantes Nantes France; ^5^ Department of Earth Sciences Durham University Durham UK; ^6^ Planetary Science Institute Tucson AZ USA; ^7^ International Research School of Planetary Sciences Università Gabriele D'Annunzio Pescara Italy; ^8^ Department of Earth Sciences Natural History Museum London UK

**Keywords:** Mars, gullies, carbon dioxide, modeling, RAMMS, Hale crater

## Abstract

Understanding the initial and flow conditions of contemporary flows in Martian gullies, generally believed to be triggered and fluidized by CO_2_ sublimation, is crucial for deciphering climate conditions needed to trigger and sustain them. We employ the RAMMS (RApid Mass Movement Simulation) debris flow and avalanche model to back calculate initial and flow conditions of recent flows in three gullies in Hale crater. We infer minimum release depths of 1.0–1.5 m and initial release volumes of 100–200 m^3^. Entrainment leads to final flow volumes that are ∼2.5–5.5 times larger than initially released, and entrainment is found necessary to match the observed flow deposits. Simulated mean cross‐channel flow velocities decrease from 3–4 m/s to ∼1 m/s from release area to flow terminus, while flow depths generally decrease from 0.5–1 to 0.1–0.2 m. The mean cross‐channel erosion depth and deposition thicknesses are ∼0.1–0.3 m. Back‐calculated dry‐Coulomb friction ranges from 0.1 to 0.25 and viscous‐turbulent friction between 100 and 200 m/s^2^, which are values similar to those of granular debris flows on Earth. These results suggest that recent flows in gullies are fluidized to a similar degree as are granular debris flows on Earth. Using a novel model for mass flow fluidization by CO_2_ sublimation we are able to show that under Martian atmospheric conditions very small volumetric fractions of CO_2_ of ≪1% within mass flows may indeed yield sufficiently large gas fluxes to cause fluidization and enhance flow mobility.

## Key Points

- We use RAMMS to back calculate initial and flow conditions of recent flows in three gullies in Hale crater
- Recent flows in gullies are fluidized to a similar degree as are wet granular debris flows on Earth
- Very small volumetric fractions of CO_2_ of ≪1% may yield sufficiently large gas fluxes to fluidize contemporary flow in Martian gullies

## 1 Introduction

Gullies are kilometer‐scale alcove‐channel‐fan systems that are present on steep slopes in midlatitude to polar regions on Mars (e.g., Conway et al., 2017; Harrison et al., 2015; Malin & Edgett, 2000). They are geologically very young features that have been active over the last few million years (De Haas et al., 2015a; De Haas et al., 2017; Johnsson et al., 2014; Reiss et al., 2004; Schon et al., 2009). Their formation has been linked to both a range of dry (e.g., Cedillo‐Flores et al., 2011; Dundas et al., 2010; Dundas et al., 2012; Dundas et al., 2015; Dundas et al., 2017; Pelletier et al., 2008; Treiman, 2003) and wet flows (e.g., Costard et al., 2002; Conway et al., 2011; Conway et al., 2015; De Haas et al., 2015b; De Haas et al., 2015c; Gulick et al., 2019; Hartmann et al., 2003; Johnsson et al., 2014; Levy et al., 2010; Lanza et al., 2010). Each of these formation processes has different implications for Mars's current and recent water cycle and, therefore, the presence of habitable environments and resources for future exploration. Some authors suggest that these processes may both have contributed to Martian gully formation but that their relative importance has varied over time by climate change forced by orbital cycles (e.g., Auld & Dixon, 2016; Conway et al., 2018a; Conway et al., 2018b; De Haas et al., 2017; Jawin et al., 2018).

Over the last decade, new flow deposits have formed within multiple gullies across Mars (e.g., Dundas et al., 2010; Dundas et al., 2017; Malin et al., 2006). Monitoring of these gullies has highlighted that these new flows form when seasonal frost is present (Diniega et al., 2010; Dundas et al., 2012; Dundas et al., 2017; Pasquon et al., 2019; Raack et al., 2015; Reiss et al., 2010). The seasonal frost may comprise both H_2_O and CO_2_, but the latter is generally more abundant and therefore generally considered a more likely candidate for the triggering of the recent flows (Diniega et al., 2010; Dundas et al., 2010; Dundas et al., 2012; Dundas et al., 2017). Accordingly, Vincendon (2015) found that most new flows were consistent with the presence of CO_2_ frost at the time of gully activity, although relatively bright gully deposits typically formed where H_2_O ice was expected but CO_2_ ice was less probable.

The recent flows that have been observed often form from a point source and are morphologically diverse (e.g., Dundas et al., 2017): they can be relatively light, neutral or dark, colorful or bland, and range from superficial deposits to 10 m‐scale topographic changes. The flows can substantially erode their channel and form terraces, transport meter‐sized boulders, form new channel segments, migrating sinuous curves, and lobate deposits (e.g., Dundas et al., 2017; Pasquon et al., 2019). An important observation is that many of these flows, despite the absence of liquid water, are more mobile and deposit on substantially lower slopes than would dry grainflows (Dundas et al., 2017). This suggests that these flows must have been fluidized; that is, something must have reduced the intergranular friction in these flows. While final flow deposits can be observed on some gully fans, morphological expressions of initial failures are generally subtle, suggesting limited initial failure volumes. Terrestrial observations indicate that mass flows can erode substantial amounts of bed material when flowing down a slope, potentially growing in size by more than a factor of 10 (e.g., Berger et al., 2011; De Haas & Van Woerkom, 2016; Hungr et al., 2005; Pérez, 2001; Schürch et al., 2011). Therefore, small‐volume initial failures may form larger mass flow deposits on gully fans. Final deposit volumes thus cannot directly inform us about initial failure volumes and conditions, which therefore remain unknown.

Multiple models for the initiation and flow conditions of recent flows in Martian gullies have recently been proposed. Slope failures initiating these flows may be triggered by deposition and sublimation of small amounts of frost on steep alcove slopes—Sylvest et al. (2016, 2018) experimentally show that under Martian conditions, sublimation of CO_2_ may trigger slope failures well below the dynamic angle of repose. Hugenholtz (2008) proposed that the highly mobile recent flows represent frosted granular flow, although Harrison (2016) later pointed out that the morphology of terrestrial examples is a poor match for Martian gullies. Alternatively, Pilorget and Forget (2016) propose a model wherein basal sublimation and rising pressure beneath a translucent slab of CO_2_ ice triggers gully activity. In this model, the enhanced gas pressures beneath the CO_2_ ice layer would fluidize the flows enhancing their mobility. However, this model does not comply with observations that there are few defrosting spots around the active gullies at low latitudes, that some activity occurs in early winter when CO_2_ condensing and gas pressure should be low, and the occurrence of recent gully activity at locations where frost abundances are low (Dundas et al., 2017). As such, Dundas et al. (2017) propose a related alternative in which gas generation occurs via two effects within a mix of sediment and CO_2_ ice tumbling down a gully: (1) the potential energy of falling material is initially converted to kinetic energy but ultimately dissipates as heat or latent heat loss (sublimation) if buffered at the CO_2_ frost point temperature; (2) eroded sediment from the shallow subsurface or unfrosted areas will be warmer than the ice and could cause additional sublimation. Mixing within the falling material will allow the generated heat to be transferred to the CO_2_ frost, causing sublimation, enhancing the gas pressure within the pores of the flowing mass leading to fluidization of the flow. Complete fluidization is not necessary to explain all recent Martian gully flows, as the gully fans are moderately steep, roughly ranging between 10° and 15° (Conway & Balme, 2016; Gulick et al., 2019), and sometimes consistent with no fluidization (Kolb et al., 2010; Conway & Balme, 2016), and it is likely that a spectrum of behaviors occurs even within individual gullies (e.g., Conway et al., 2018b; De Haas et al., 2017).

These processes represent a potential source of fluidization unknown to terrestrial mass movements. Although the proposed processes are not necessarily mutually exclusive, they have a few common denominators. An initial slope failure in the gully alcove becomes a moving high‐density mass flow, wherein some sort of fluidization occurs enhancing flow mobility in the absence of liquid water. Moreover, the flows are capable of eroding bed and/or bank material, causing the flow to grow in volume as it travels down the gully.

At present, we do not know the initial conditions and flow conditions of these flows. For example, we do not know their initial failure volume, degree of fluidization, and flow parameters such as flow velocity, flow depth, and erosion rate. Deciphering these conditions may inform us of the climatic conditions, that is, amounts of CO_2_ that need to precipitate from the atmosphere to trigger and sustain these recent flows. The lack of a terrestrial analog to compare to recent flows in Martian gullies inhibits resolving these climatic conditions through comparative studies. Therefore, numerical modeling of these flows might be the key, although many terrestrial mass flow models assume liquid water within the flows and are therefore not applicable. Only a few studies have used numerical mass flow models to reconstruct initial and flow conditions in Martian gullies so far (Kolb et al., 2010; Mangold et al., 2010; Pelletier et al., 2008). However, the numerical model employed by these studies does not directly use Martian gravity, does not include entrainment, and requires an input hydrograph rather than an initial slope failure such as observed for the recent flow deposits on Mars, which are all critical parameters for accurately resolving flow and initiation conditions of recent flows in Martian gullies.

**Figure 1:**
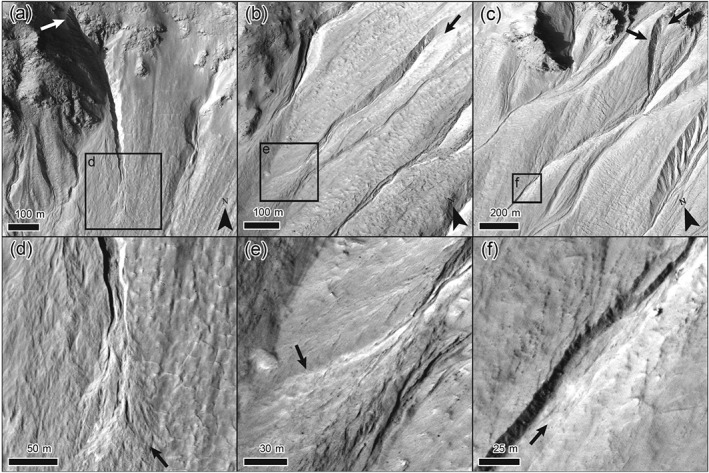
Studied gullies. (a) Gully 69. The white arrow indicates the location of the release area. (b) Gully 9. The black arrow indicates the release area. (c) Gully 26. The black arrows indicate the release areas. (d) Detail of new deposit in gully 69. (e) Detail of new deposit in gully 9. (f) Detail of new deposit in gully 26. Black arrows in panels (d)–(f) indicate the new deposits. See supporting information movies for more details. HiRISE image ESP_038218_1445.

**Figure 2:**
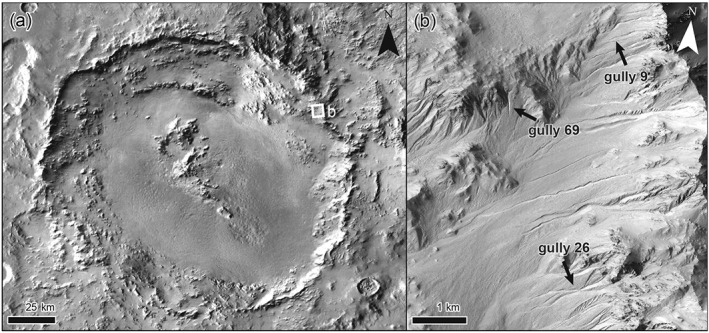
Hale crater and study site overview. (a) Hale crater, centered near 35.8°S, 325.5°E. Thermal emission imaging system (THEMIS) daytime infrared mosaic. (b) Overview of the studied gullies, see supporting information Figure S1 for an overview of all observed gully activity and Figure 1 for more details of the study sites. HiRISE image ESP_038218_1445.

We modified the RAMMS (RApid Mass Movement Simulation) debris flow and avalanche model (Christen et al., 2012) to permit its use under Martian conditions, and then used this model to back calculate and infer initial and flow conditions in recent flows in Martian gullies. In particular, we aim to (1) constrain initial failure conditions; (2) determine flow properties including flow velocity and flow depth; (3) define the rate of fluidization within the flows; and (4) test whether CO_2_ sublimation could account for the inferred fluidization. To do so, we back calculate three recent flows in Hale crater (Figures 1 and 2).

## 2 Study Sites

Hale crater is an oblate impact crater with a N‐S diameter of 125 km and E‐W diameter of 150 km and is located in the southern hemisphere at 35.7°S, 323.6°E (Figure 2). The crater contains a central peak and multiple wall terraces (e.g., Melosh, 1989), and is estimated to have formed around 1 Ga (Jones et al., 2011). Gullies are abundant on both the central peak and crater walls on multiple slope orientations (e.g., Dickson et al., 2007; De Haas et al., 2017; Kolb et al., 2010; Reiss et al., 2009; Reiss et al., 2011). Numerous new flow deposits have formed in Hale crater gullies over the last decade (Dundas et al., 2012; Dundas et al., 2015; Dundas et al., 2017; Kolb et al., 2010; McEwen et al., 2007).

On a ∼13‐km‐long stretch of the eastern wall of the crater (Figure 2) we observe activity in over 40 gully systems between 12 March 2007 (HiRISE image PSP_002932_1445) and 21 September 2014 (HiRISE image ESP_038218_1445) (supporting information Figure S1). These flows generally have a bright appearance, were typically initiated on the steep slopes of the gully catchments, and have various runouts, depositing in the medial to distal parts of the gully systems. Kolb et al. (2010) observed that the recent flows in this area generally form thin terminal deposits, with an estimated thickness of a few decimeters at maximum. We select three sites for detailed numerical modeling (Figure 1—systems 9, 26, and 69 in Figure S1) that (1) have an identifiable release area; (2) have a long travel distance; (3) have a well‐defined and identifiable depositional area; and (4) are of considerable size, eroding material in the upper parts of the flow route and transporting multiple boulders (see supporting information movies).

The alcoves of the gullies in the study sites are broadly covered by a thick layer of pasted‐on terrain believed to consist of fine‐grained sediment and water ice (e.g., Conway & Balme, 2014). This material is usually interpreted as a latitude‐dependent mantle located on sloping terrain formed from airfall of ice nucleated on dust (e.g., Head et al., 2003; Milliken et al., 2003; Mustard et al., 2001) but may also have been (partly) reworked by glaciation and be predominantly glacial in origin (cf. Conway et al., 2018a). One of our study sites, gully 9 (Figure 1b), solely cuts into this pasted‐on terrain and therefore probably largely consists of fine‐grained materials, while gullies 26 and 69 (Figures 1a and 1c) have source areas consisting of both bedrock and pasted‐on terrain and may thus potentially transport larger clasts.

## 3 Materials and Methods

### 3.1 RAMMS

We perform our simulation using the RAMMS model (Christen et al., 2012). RAMMS was originally developed to model snow avalanches (e.g., Christen et al., 2010a; Fischer et al., 2012) but has also been applied to other types of wet and dry mass movements including landslides (e.g., Christen et al., 2012; Fan et al., 2017), rock avalanches (e.g., Allen et al., 2009; Schneider et al., 2010), and debris flows (e.g., Frank et al., 2015; Hussin et al., 2012). RAMMS is a 2‐D depth‐averaged continuum model capable of simulating flow velocities, flow heights, impact pressures, flow path, and runout over 3‐D topography. The model is typically used for hazard analysis and hazard mapping studies in Alpine areas (e.g., Schraml et al., 2015), in regions with steep source areas similar in morphometry to Martian gullies.

The model is suitable for our purposes because it does not require the definition of an interstitial fluid. Instead, in RAMMS flow dynamics are defined through frictional resistance. The model divides the frictional resistance into two parts: a dry‐Coulomb type friction *μ* (‐) that scales with the normal stress and a velocity‐squared drag or viscous‐turbulent friction *ξ* (m/s^2^). The frictional resistance *S* (Pa) is then

(1) $$S=\mu N+\frac{\rho gu^{2}}{\xi }\;\text{with}\;N=\rho h\left[g\cos\theta +\kappa u^{2}\right]$$

where *ρ* is the flow density $\left({\text{kg/m}}^{3}\right)$, *g* is the gravitational acceleration (3.72 m/s^2^ on Mars), *h* is the flow height (m), *θ* is the slope angle, *u* is the flow speed parallel to the surface (m/s), and *κ* is the terrain curvature in the direction of flow (m^−1^). The friction coefficients are responsible for the behavior of the flow, where the resistance of the solid phase *μ* dominates the flow close to stopping, while the viscous‐turbulent friction *ξ* dominates when the flow is running quickly.

Mass flows, such as dry granular flows and debris flows, can erode substantial amounts of bed material during flow (e.g., Berger et al., 2011; Schürch et al., 2011; Theule et al., 2015), increasing flow volume and thus affecting flow behavior and runout. The processes of bed erosion in mass flows are poorly understood and therefore difficult to predict (e.g., De Haas & Van Woerkom, 2016; Schürch et al., 2011). The erosion module of RAMMS is based on field observations in the Illgraben torrent, Switzerland, which indicate that the depth of erosion increases linearly with basal‐shear stress *τ* [Pa] (Frank et al., 2015; Schürch et al., 2011) at a specific erosion rate (Berger et al., 2011). The same observations show that relatively small debris flows do not always erode sediment (Berger et al., 2010; Schürch et al., 2011), which indicates that mass flows only erode bed material above a critical shear stress *τ*_*c*_ [Pa].

Basal shear stress is defined as

(2) τ=ρghsin(θ)

where *θ* is the channel bed slope. The maximum erosion depth *e*_*m*_ is calculated as

(3) $$e_{m}=0\;\text{for}\;\tau <\tau_{c}$$

(4) $$e_{m}=\frac{dz}{d\tau }(\tau -\tau_{c})\;\text{for}\;\tau \geq \tau_{c}$$

in this scheme sediment is entrained when *τ* ≥ *τ*_*c*_ at rate $\frac{dz}{d\tau }$ [m/Pa] until the potential erosion depth *e*_*m*_ is reached. We assume that an erodible substrate is available over the full length of the studied gully flows.

We let flows initiate as shallow landslides (slope failures) of a given area and depth. We assume that the initial landslide instantaneously starts flowing downslope and is not formed by a series of retrogressive failures. Flows in RAMMS stop once the initial momentum has declined below a user‐defined criterion, which is defined as a fraction of the initial momentum (set to 10% in our simulations) (Bartelt et al., 2017).

### 3.2 Choice of Parameters

To constrain the plausible range of initial slope failure volumes and combinations of dry‐Coulomb and viscous‐turbulent frictions, we explore the parameter range shown in Table 1. The area of the initial slope failures was constrained from the HiRISE imagery, and we vary the depth of the initial slope failures that initiate the mass flows between 0.5 and 2.0 m. We choose this range because we need at least an initial release depth (i.e., thickness of the initial failure zone) of ∼0.5 m to enable flow initiation and because slope values deeper than 2.0 m are unrealistic given the subtle changes in observed release areas and the likelihood of ground ice with overlying lag in the Martian midlatitudes that limits the failure depth. For the friction parameters we explore a range of values observed in a wide range of terrestrial mass flows (e.g., Allen et al., 2009; Hussin et al., 2012; Frank et al., 2015; Frank et al., 2017; Schneider et al., 2010). The dry‐Coulomb friction ranges from 0.05 to 0.50. The viscous‐turbulent friction ranges between 100 and 1,000 m/s^2^, where values between 100 and 200 m/s^2^ generally correspond to solid‐dominated flows and values between 200 and 1,000 m/s^2^ correspond to fluid‐like flows.

**Table 1:** RAMMS Model Parameters

Parameter	Value	
*Variable parameters*		
Slope failure depth	0.5–2.0 m	0.5‐m intervals
Coulomb friction	0.05–0.50	0.05 intervals
Viscous‐turbulent friction	100–900 m/s^2^	100‐m/s^2^ intervals
*Fixed parameters*		
Gravitational acceleration	3.72 m/s^2^	
Stopping momentum	10%	
Flow density	1,500 kg/m^3^	
Bed‐material (erosion) density	2,000 kg/m^3^	
Erosion rate	0.025 m/s	
Potential erosion depth	0.1 m/kPa	
Critical shear stress	1 kPa	

*Note.* RAMMS = RApid Mass Movement Simulation.

To simulate the observed dry flows we assume a fixed flow density of 1,500 kg/m^3^, corresponding to a flow porosity of 0.5, basaltic particles with a density of ∼3,000 kg/m^3^, and CO_2_ gas in between the pores (cf. Pilorget & Forget, 2016). We further assume that the material that is eroded from the bed has a lower porosity and therefore a bulk density of 2,000 kg/m^3^. Bed entrainment by mass flows on Earth and Mars is poorly understood due to a lack of observations (e.g., Berger et al., 2010; De Haas & Van Woerkom, 2016; Iverson et al., 2011; Schürch et al., 2011; Theule et al., 2015). Therefore, we base our parameters of erosion rate, potential erosion depth per unit of shear stress, and critical shear stress for erosion on the only measurements available (Berger et al., 2011; Frank et al., 2015; Schürch et al., 2011). We realize that there are uncertainties related to transferring these values based on terrestrial measurements to Mars and that different parameter values may yield different results (see Frank et al., 2017, for an extensive sensitivity analysis). The key point here is that inclusion of bed erosion is critical to reproduce the observed recent flows on Mars that start from relatively small initial slope failures. Moreover, the flows are observed to erode material while flowing through gullies, showing the importance of including this process. Note that we do not consider such low values of Coulomb friction to represent any realistic physical process, but merely necessary parameter choices to match observations.

### 3.3 Parameter Fitting

We back calculate combinations of initial flow volume, dry‐Coulomb friction, and viscous‐turbulent friction by comparing model outputs to three types of observations. These are (1) total travel distance, (2) erosion distance (the distance from the release area to the most downstream point where erosion is observed), and (3) the flow depth estimated from boulder diameters transported in the flows.

We define the travel distance error as the distance between the simulated and observed travel distance (e.g., Mergili et al., 2017). Similarly, we calculate the erosion distance error, which is the distance between the simulated and observed erosion distance. We combine both into a combined error, where we attribute a weight of two thirds to the travel distance error and one third to the erosion distance error. We attribute more weight to the travel distance error, because the erosion distance error partly depends on the travel distance error and partly because of the unknown erosion parameters that we based on terrestrial values.

### 3.4 Elevation Model Production and Filtering

We produced a digital terrain model (DTM) for the study site from two HiRISE stereo images; PSP_002932_1445 and PSP_003209_1445. The DTM was produced with the software packages ISIS3 and SocetSet following the workflow described by Kirk et al. (2008). The relative vertical precision of the DTM is 0.11 m, calculated as maximum resolution/5/tan (convergence angle) (cf.Kirk et al., 2008). To minimize the influence of small errors and uncertainties in the DTM, we applied a 3 × 3 low‐pass filter (cf. Kolb et al., 2010) and thereafter filled sinks via standard GIS procedures.

## 4 Results

### 4.1 Observed Release and Flow Conditions

#### 4.1.1 Gully 69

**Figure 3:**
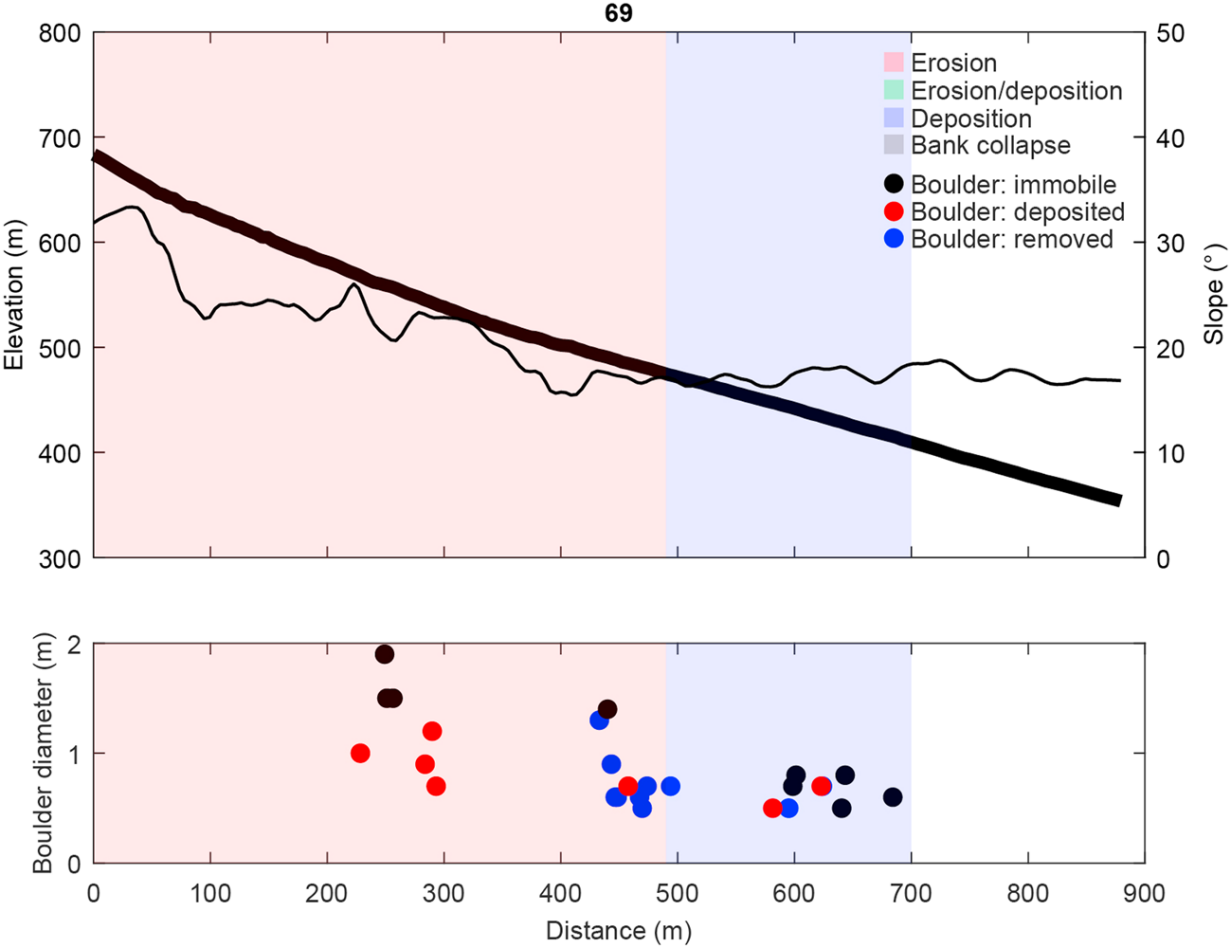
(top) Channel bed elevation, channel bed slopes, observed net erosion, net deposition, and bank collapse along the studied recent flow, and (bottom) the diameter and location of mobilized (deposited or removed) and immobile boulders for gully 69 (Figure 1a). Immobile boulders have not moved between the 2007 and 2014 images, removed boulders were present on the 2007 image but were removed on the 2014 image, and deposited boulders were not present on the 2007 image but appeared on the 2014 image. See supporting information Figure S2 for boulder locations. The thick black line corresponds to the elevation, and the thin black line corresponds to the slope.

The recent flow in gully 69 has a total travel distance of 700 m (Figure 3 and supporting information movies). Over its first 600 m of travel the flow was channelized, while the final 100 m of flow was on an unchannelized depositional fan. The flow initiated at an elevation of 680 m, and reached down to an elevation of 410 m. Near its initiation point channel slopes are around 35°, and channel slopes are 16–18° in the deposition zone. Net channel bed erosion was observed in the first 500 m of flow, after which the flow was net depositing in the final 200 m. The transition from net erosion to net deposition occurred where the channel bed slope dropped below 16–18°, with net erosion on the steeper slopes upstream of the erosion‐deposition transition point. In total, 26 boulders were present in the flow path, of which 17 were mobilized by the flow. The diameter of the mobilized boulders is generally <1 m and decreases downstream.

#### 4.1.2 Gully 9

**Figure 4:**
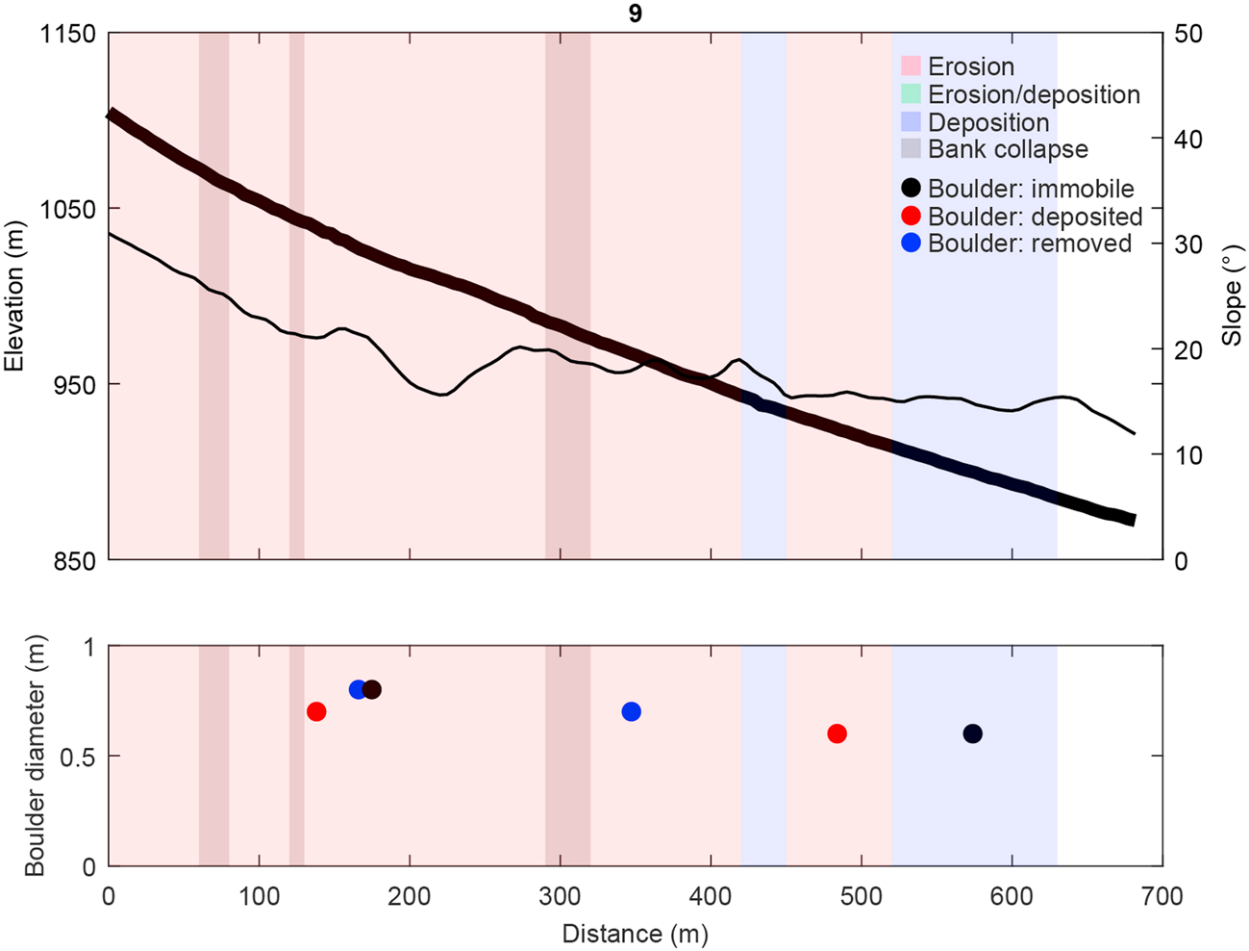
(top) Channel bed elevation, channel bed slopes, observed net erosion, net deposition and bank collapse along the studied recent flow, and (bottom) the diameter and location of mobilized (newly deposited or removed) and immobile boulders for gully 9 (Figure 1b). Immobile boulders have not moved between the 2007 and 2014 images, removed boulders were present on the 2007 image but were removed on the 2014 image, and deposited boulders were not present on the 2007 image but appeared on the 2014 image. See Figure S2 for boulder locations. The thick black line corresponds to the elevation, and the thin black line corresponds to the slope.

The recent flow in gully 9 has a travel distance of 630 m (Figure 4). The flow originates from a shallow landslide in the catchment headwaters at an elevation of 1,100 m and terminates at an elevation of 880 m (Movies S1–S8). The flow is channelized for about 530 m and flows over an unchannelized depositional fan for the final 100 m. Channel bed slopes are approximately 30° in the initiation area and decrease to approximately 15° at the flow terminus. Only six boulders are present in the flow path, likely as a result of the gully alcove solely cutting into pasted‐on material. These boulders are relatively small having diameters ranging from 0.6 to 0.8 m. In total, four of these boulders have been mobilized by the flow, where the boulders in the net erosional zone are generally mobile, while the one boulder in the net depositional zone was immobile. The flow was net erosional for the first 510 m along its flow path, with a minor net depositional zone around a distance of 430 m. Bank collapse occurred near the catchment headwaters and around a distance of 300 m. Over its final 120 m of travel the flow was net depositional. The erosion‐deposition transition occurred around a channel bed slope of 15–16°.

#### 4.1.3 Gully 26

**Figure 5:**
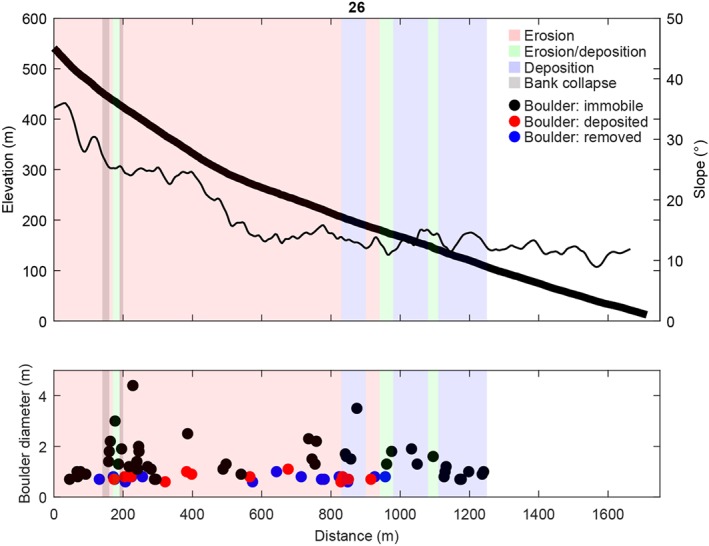
(top) Channel bed elevation, channel bed slopes, observed net erosion, net deposition, and bank collapse along the studied recent flow, and (bottom) the diameter and location of mobilized (newly deposited or removed) and immobile boulders for gully 26 (Figure 1c). Immobile boulders have not moved between the 2007 and 2014 images, removed boulders were present on the 2007 image but were removed on the 2014 image, and deposited boulders were not present on the 2007 image but appeared on the 2014 image. See Figure S2 in the supporting information for boulder locations. The thick black line corresponds to the elevation, and the thin black line corresponds to the slope.

The travel distance of the recent flow in gully 26 was ∼1,250 m (Figure 5). The flow originates from two release areas, with the second release area being ∼100 m downstream of the first (Movies S1–S8). The flow path was fully channelized, with the flow terminating in the channel ∼200 m upstream of the gully fan. The flow initiated at an elevation of 530 m and terminated at an elevation of 110 m. Channel bed slopes in the initiation area were ∼35° and channel bed slopes in the final depositional area were approximately 13°. A total of 74 boulders were present in the flow path, of which 27 were mobilized by the flow. In general, boulders with a diameter of 1 m or smaller were mobilized by the flow, while larger boulders were immobile. In the final 200 m of the flow, roughly corresponding to the net depositional part of the flow, all boulders were immobile. In the first 800 m of the flow there was net erosion, with observed bank collapses near 200 m travel distance. From 800–1,000 m net erosional and net depositional reaches were alternating, and from 1,000‐m travel distance onward the flow was net depositional. The erosion‐deposition point occurs at around a channel bed slope of 13–14°.

### 4.2 Back Calculation of Release Depth and Friction Parameters

#### 4.2.1 Gully 69

**Figure 6:**
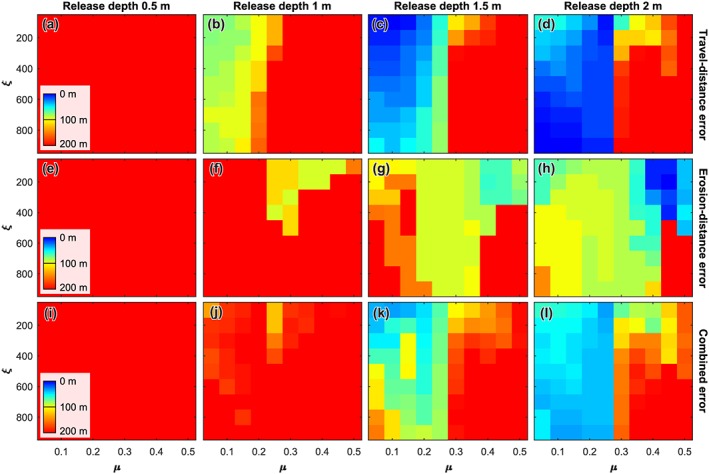
Model performance for gully 69. (a–d) Travel distance error for release depths of 0.5, 1.0, 1.5, and 2.0 m, respectively. (e–h) Erosion distance error for release depths of 0.5, 1.0, 1.5, and 2.0 m, respectively. (i–l) Combined error of travel distance and erosion distance for release depths of 0.5, 1.0, 1.5, and 2.0 m, respectively. To obtain the combined error we appoint a weight of two thirds to the travel distance error and one third to the erosion distance error.

For gully 69 there are no flows with a release depth of 0.5 and 1.0 m that are able to reproduce the observed travel and erosion distance (Figure 6). For release depths of 1.5 and 2 m, the travel distance is roughly reproduced by flows with a dry‐Coulomb friction (*μ*) smaller than 0.25. For flows with a release depth of 1.5 m the erosion distance is best reproduced for flows with intermediate dry‐Coulomb frictions around 0.25 over a wide range of viscous‐turbulent friction values. For these flows the erosion distance is roughly underestimated by 100 m. A similar trend is visible for flows with a release depth of 2 m, although the range of friction parameter combinations for which the erosion distance is reasonably reproduced is larger. The erosion distance is well‐reproduced by flows with large dry‐Coulomb friction and small viscous‐turbulent friction, but for these flows the runout distance is poorly reproduced. The combined error shows that flows with a release depth of 1.5 m, small viscous‐turbulent friction of 300 m/s^2^ or less and a dry‐Coulomb friction of 0.25 or less yield realistic results. For flows with a release depth of 2 m, realistic results are produced for nearly all flows with a dry‐Coulomb friction of 0.25 or less.

#### 4.2.2 Gully 9

**Figure 7:**
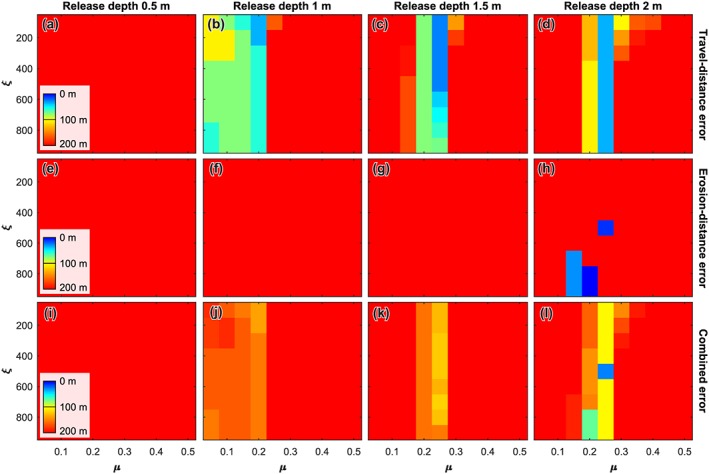
Model performance for gully 9. (a–d) Travel distance error for release depths of 0.5, 1.0, 1.5, and 2.0 m, respectively. (e‐h) Erosion distance error for release depths of 0.5, 1.0, 1.5, and 2.0 m, respectively. (i–l) Combined error of travel distance and erosion distance for release depths of 0.5, 1.0, 1.5, and 2.0 m, respectively. To obtain the combined error we appoint a weight of two thirds to the travel distance error and one third to the erosion distance error.

The smallest release depth that is capable of accurately reproducing the travel distance of the recent flow in gully 9 is 1.0 m (Figure 7). For flows with a release depth of 1.0 m the travel distance is reproduced by flows with a dry‐Coulomb friction of 0.2 and a viscous‐turbulent friction of 100–200 m/s^2^. For a release depth of 1.5 m travel distance is well‐reproduced by flows with a dry‐Coulomb friction of 0.2 and a viscous‐turbulent friction of 100–500 m/s^2^. The erosion distance is underestimated for all flows with a release depth of <1.5 m. Flows with a release depth of 2 m and a dry‐Coulomb friction of 0.25 accurately reproduce the travel distance for the full range of simulated viscous‐turbulent friction values of 100–900 m/s^2^. For these flows, the erosion distance is reproduced for a viscous‐turbulent friction value of 500 m/s^2^.

#### 4.2.3 Gully 26

**Figure 8:**
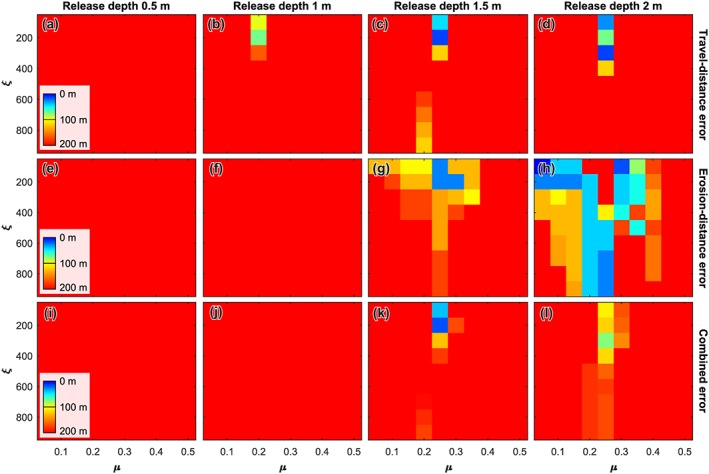
Model performance for gully 26. (a–d) Travel distance error for release depths of 0.5, 1.0, 1.5, and 2.0 m, respectively. (e–h) Erosion distance error for release depths of 0.5, 1.0, 1.5, and 2.0 m, respectively. (i–l) Combined error for release depths of 0.5, 1.0, 1.5, and 2.0 m, respectively. To obtain the combined error we appoint a weight of two thirds to the travel distance error and one third to the erosion distance error.

The runout distance of the recent flow in gully 26 is only reproduced for release depths of 1.5 and 2.0 m (Figure 8). For a release depth of 1.5 m, model runs with a dry‐Coulomb friction of 0.25 and a viscous‐turbulent friction of 100–200 reasonably reproduce the observed travel distance, with a viscous‐turbulent friction of 200 m/s^2^ yielding the best results. The location of the erosion distance is also best reproduced by flows with a dry‐Coulomb friction of 0.25 and a viscous‐turbulent friction of 200 m/s^2^, causing this combination of parameters to yield the best model results. For flows with a release depth of 2 m, runout distance is reasonably reproduced for flows with a dry‐Coulomb friction of 0.25 and viscous‐turbulent friction ranging from 100–300 m/s^2^, where a value of 300 m/s^2^ yields the best result. The location of the erosion‐deposition transition is correctly reproduced by model runs with a small dry‐Coulomb and viscous‐turbulent friction, a dry‐Coulomb friction of 0.20–0.25 and a large viscous‐turbulent friction, or a dry‐Coulomb friction of 0.30–0.35 and a small viscous‐turbulent friction. For a dry‐Coulomb friction of 0.25 and a viscous‐turbulent friction of 100–300 m/s^2^, the best‐fit values for travel distance, the erosion distance is poorly reproduced—erosion occurs too far downstream, although in local patches only. As a result, the combined error for these combinations of friction parameters, reproducing the observed flows well, is relatively large.

### 4.3 Most Plausible Flow Conditions

**Figure 9:**
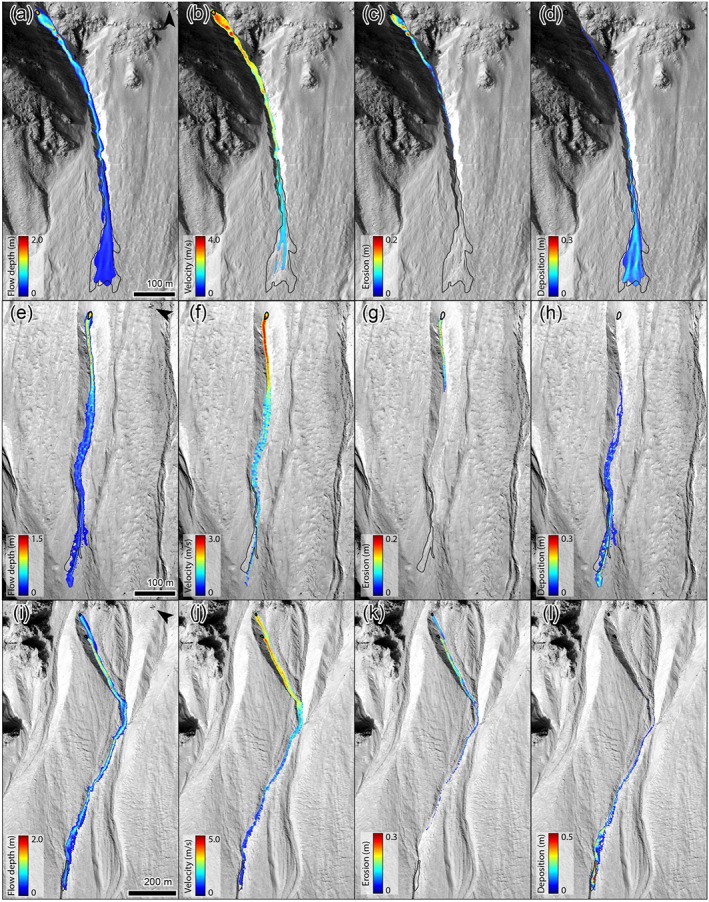
Simulated flow conditions for plausible simulations of the study sites. (a–d) Maximum flow depth, maximum flow velocity, maximum erosion depth, and final deposit thickness for a flow with a release depth of 1.5 m, a dry‐Coulomb friction of 0.10, and a viscous‐turbulent friction of 100 m/s^2^ in gully 69. (e–h) As above for a flow with a release depth of 1.0 m, a dry‐Coulomb friction of 0.20, and a viscous‐turbulent friction of 200 m/s^2^ in gully 9. (i–l) As above for a flow with a release depth of 1.5 m, a dry‐Coulomb friction of 0.25, and a viscous‐turbulent friction of 200 m/s^2^ in gully 26. The black outlines indicate the observed release and depositional areas. HiRISE image ESP_038218_1445.

**Figure 10:**
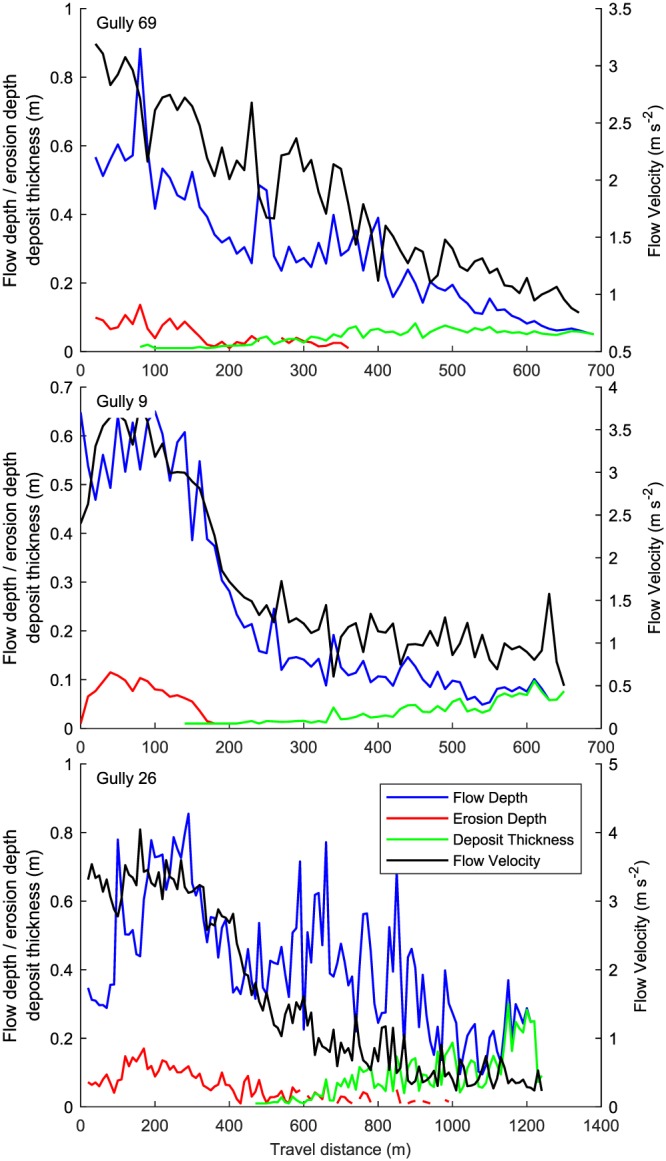
Simulated mean cross‐channel flow characteristics for the flows shown in Figure 9. (top) Gully 69, (middle) gully 9, and (bottom) gully 26.

Here we summarize the initial and flow conditions of the most plausible flows for each of the three studied flows. We define the most plausible flow as the friction parameter combination yielding plausible travel and erosion distances for the smallest release depth possible, thereby matching the observed subtle release depths. As such, we find that the most plausible flow for gully 69 has a release depth of 1.5 m, a dry‐Coulomb friction of 0.10, and a viscous‐turbulent friction of 100 m/s^2^. For gully 9 we find a release depth of 1.0 m, a dry‐Coulomb friction of 0.2, and a viscous‐turbulent friction of 200 m/s^2^. For gully 26 we find a release depth of 1.0 m, a dry‐Coulomb friction of 0.25, and a viscous‐turbulent friction of 200 m/s^2^ (Figures 9 and 10).

The initial release volumes for gullies 69, 9, and 26 are 141, 111, and 201 m^3^, respectively (Table 2). All flows grow in size by eroding sediment, bulking by a factor 2.9, 2.4, and 5.4 for gullies 69, 9, and 26, respectively. Simulated flow depths and velocities decrease downstream for all flows (Figures 9 and 10). Initial mean flow velocities are 3–4 m/s, and decrease to <1 m/s at the flow terminus. Initial mean flow depths are ∼0.6 m and decrease down to 0.5–0.1 m at the flow terminus. Entrainment occurs in the upper parts of the flow paths, where flow depths are relatively large and the channel is steep (Figures 3, 4, 5). Simulated mean entrainment depths are restricted, in the order of ∼0.1 m. Material is mostly deposited in the lower reaches of the flows, and deposit thicknesses are generally largest near the flow terminus. The simulated mean deposit thicknesses are around 0.1–0.2 m at maximum.

**Table 2:** Best Fit Model Run Initiation and Flow Characteristics

Flow characteristic	69	9	26
Release depth (m)	1.5	1.0	1.5
Release volume (m^3^)	141	111	201
Eroded volume (m^3^)	274	152	876
Final volume (m^3^)	415	263	1,077
Bulking factor (‐)	2.9	2.4	5.4
Dry‐Coulomb friction (‐)	0.1	0.20	0.25
Viscous‐turbulent friction (m/s^2^)	100	200	200

### 4.4 Boulder Transport

We observe mobile and immobile boulders in the studied recent gully flows. To evaluate the models' performance to predict boulder transport we therefore compare boulder diameter to the locally simulated maximum flow depth for the most plausible flow conditions described above.

On Earth, it is commonly assumed that boulder transport on slopes of ∼6° or steeper occurs by mass flows (Stock & Dietrich, 2003). Although we are unaware of any work explicitly resolving the critical flow conditions for boulder entrainment and transport in such mass flows, observations from fluvially dominated streams tell us that boulders are often relatively mobile in steep torrents (e.g., Vollmer & Kleinhans, 2007). This is the combined result of the large exposure surface of such boulders that therefore experience large drag forces (e.g., Carling & Glaister, 1987), the reduced specific density of boulders that are fully submerged compared to those that partly protrude the water surface (e.g., Lamb et al., 2008; Vollmer & Kleinhans, 2007), and the effects of submergence – that is, particles are more easily entrained with increased submergence because the area of a particle affected by the flow, and thus the total flow force, increases with submergence (e.g., Vollmer & Kleinhans, 2007). As a first estimate we therefore assume that boulders in Martian gullies may be entrained and transported once flow depth equals or exceeds the boulder diameter. Segregation effects are also likely to be significant.

**Figure 11:**
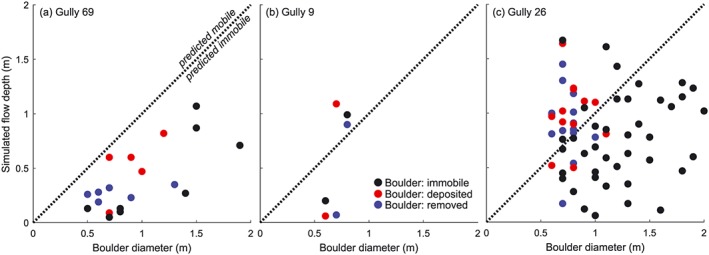
Observed boulder diameter versus simulated flow depth for the plausible simulations (Figure 9). (a) Gully 69: release depth = 1 m, *μ* = 0.10, *ξ* = 100 m/s^2^. (b) Gully 9: release depth = 1.0 m, *μ* = 0.20, *ξ* = 200 m/s^2^. (b) Gully 26: release depth = 1.5 m, *μ* = 0.25, *ξ* = 200 m/s^2^.

Via this approach we are able to accurately reproduce the observed boulder movement in gully 26 (Figure 11). For gully 69, however, the flow depth is always shallower than the boulder size, while for gully 9 boulder mobility and immobility are also poorly reproduced. This may be explained by (1) local maxima in flow depth not resolved by the model that caused boulder movement, (2) the underestimation of erosion distance by the model for gullies 69 and 9 in turn leading to underestimation of flow depth, or (3) an unknown additional process that contributes to boulder movement.

## 5 Discussion

### 5.1 Recent Flows in Martian Gullies

#### 5.1.1 Initial and Flow Conditions

Observations of the recent flows in Hale crater indicate that they are generated by restricted release areas, erode bed and bank sediments when traversing down the gully and form deposits with restricted thickness estimated to be in the order of a few decimeters at maximum (e.g., Kolb et al., 2010). We are able to successfully reproduce these observed initial and flow conditions through simulations with RAMMS (Table 2). We back calculate that the three studied recent flows in Hale require minimum release depths of 1.0–1.5 m and initial release volumes of 100–200 m^3^. These flows grow in size by a factor ∼2.5–5.5 by entraining bed materials. Entrainment is necessary to meet the observed travel distance and deposits—in the absence of entrainment and bulking the flows have a travel distance that is too short. The reproduced mean cross‐channel flow velocities are in the range of 3–4 m/s near the release area where channel slopes are large and decrease to ∼1 m/s near the flow termination point (Figures 9 and 10). Mean cross‐channel flow depths generally decrease from 0.5–1 m near the release area to 0.1–0.2 m near the flow terminus. The mean cross‐channel erosion depth and deposition thickness are generally subtle, in the order of 0.1–0.2 m, in line with observations of limited erosion depth and deposit thickness.

The back‐calculated flow velocities for the recent flows in Hale crater are in the range of flow velocities obtained semiempirically for flows in gullies forming on the lee side of the Russell crater dune: of 1–7 m/s obtained from levee asymmetry (Mangold et al., 2003) and 5–7 m/s inferred from the Manning equation (Jouannic et al., 2012). The only two previous studies that modeled recent flows in Martian gullies with a 2‐D depth‐averaged model running over 3‐D topography were performed with FLO‐2D (Kolb et al., 2010; Pelletier et al., 2008), wherein entrainment is neglected and flows are released through a hydrograph rather than a shallow landslide. For a recent flow in Penticton crater with a travel distance of 1,250 m and similar release and depositional slopes to those of the flows in Hale crater studied here, Pelletier et al. (2008) back calculated a flow volume of 2,500 m^3^, a maximum flow depth of 1.0 m (excluding the proximal flow region), and a maximum flow velocity of ∼8 m/s. Kolb et al. (2010) back‐calculated flow conditions of two recent flows in the same study area in Hale crater as studied here, simulating wet sediment‐rich flows. They found flow volumes of 2,400 and 3,200 m^3^, and mean channel velocities of 2–3 and 3–4 m/s, respectively. Inferred deposit thicknesses of 1‐ to 2‐m thick for both flows. Observed flow volumes were 385 and 940 m^3^, respectively, and thus almost an order of magnitude smaller than those inferred from the model, and observed deposit thicknesses of a few decimeters at maximum were also overestimated by an order of magnitude. By using an initial slope failure and including bed entrainment and flow bulking, we are thus able to more accurately reproduce the observed initial, flow and depositional conditions of recent flows in Martian gulllies, highlighting the importance of these processes in recent gully flows. The limited back‐calculated release and erosion volumes confirm previous findings that Martian gullies must have formed by the combined effect of tens of thousands of flows to obtain their current morphology (e.g., De Haas et al., 2015b; Dundas et al., 2015).

#### 5.1.2 Erosion‐Deposition Transition

The studied recent flows in Hale crater changed from erosion to deposition at slopes ranging from 13–14° in gully 26, 15–16° in gully 9 to 16–18° in gully 69. Pelletier et al. (2008) pointed out that dry granular flows typically deposit on slopes of around 21°, but this is very far from values observed in experiments where angles >30° are typical for natural materials. Presumably much of this difference is due to the slope angle changing significantly during the time taken for the flow to stop.

The static angle of repose on Mars is currently under debate. Kleinhans et al. (2011) claim that the static angle of repose on Mars is a few degrees larger than that on Earth as a result of the lower gravity, while experimental observations from Balmforth and McElwaine (2018) and observations on Mars (Atwood‐Stone & McEwen, 2013) suggest no change. Nevertheless, Balmforth and McElwaine (2018) show that there is a wide range of angles over which failure and erosion can occur even in a completely controlled setting. In the natural, uncontrolled, environment there are additional factors affecting erosion, including flow depth, flow velocity, composition of the bed material, and composition of the flow (e.g., De Haas & Van Woerkom, 2016).

The critical slopes for erosion observed in the recent flows in Hale crater are in the range of those observed for terrestrial debris flows. On Earth, critical slopes for debris flow erosion in natural torrents range from ∼9° in two torrents in the French Alps (Theule et al., 2015), 8–12° (Hungr et al., 1984) or 12–15° (Guthrie et al., 2010) for debris flows in British Columbia, 16° on the Kamikamihora fan in Japan (e.g., Okuda & Suwa, 1984; Takahashi, 2009) and 19° for hillslope debris flows in Iceland (Conway et al., 2010).

In summary, the observed critical erosion slope in Hale crater falls within the range observed for terrestrial wet sediment gravity flows, but is lower than would be expected by typical dry natural materials with angles of >30° (*μ*>0.58). Since there is no likely source of liquid water for the studied Martian flows, this strongly suggests that another mechanism is at work; likely partial fluidization by CO_2_ sublimation. The relatively large critical slope for erosion in Martian gullies may also be the result of the absence of liquid water in the bed. Most terrestrial debris flows are triggered during high‐intensity rainfalls, which increases the water content in the bed before passage of a debris flow. This increases the erodibility of the channel bed as a result of increased pore pressure (Iverson et al., 2011; Reid et al., 2011). As the studied Martian flows are almost certainly generated under dry conditions, this may thus also explain their critical erosion slopes at the upper end of the terrestrial spectrum, although we do not know how solid CO_2_ in the channel bed may affect critical slopes for erosion.

#### 5.1.3 Fluidization by CO_2_ Sublimation

**Figure 12:**
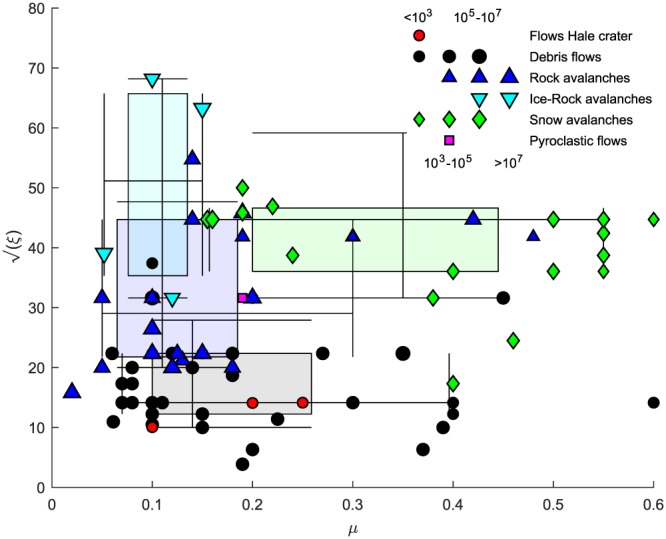
Vöellmy friction parameter combinations of the recent flows in Hale crater and terrestrial values obtained from back calculations with RAMMS and DAN(3D) from a wide range of debris flow, rock avalanche (also containing debris avalanches and landslides), ice‐rock avalanches, snow avalanches, and a pyroclastic flow. See Appendix A for source data of the terrestrial values. The shaded boxes are two‐dimensional boxplots of all data per flow type, where boxes indicate quartiles, line crossings indicate the median, and whiskers indicate the 10th and 90th percentiles. See Table A1 for source data. RAMMS = RApid Mass Movement Simulation.

We are able to reproduce the observed flow properties for flows with a dry‐Coulomb friction in the range of 0.1–0.25 and a viscous‐turbulent friction of 100–200 m/s^2^ (Table 2). These friction values are similar to those found by back calculation of a wide range of terrestrial debris flows, while they differ from the friction values found by back calculation of a wide range of rock avalanches, ice‐rock avalanches, snow avalanches, and a pyroclastic flow that have larger dry‐Coulomb and viscous‐turbulent friction values (Figure 12). Note that the frictional resistance of the flows increases with increasing values of dry‐Coulomb friction (*μ*) and decreases for increasing numbers of viscous‐turbulent friction (*ξ*), as given by equation (1). Small viscous‐turbulent friction values (≤200 m/s^2^) are typically found for granular debris flows while relatively large viscous‐turbulent friction values are typically found for more viscous and muddy debris flows (>200 m/s^2^) (Bartelt et al., 2017). This is consistent with observations of recent gully flows on Mars, which are often hypothesized to be granular flows fluidized by gas pressure induced by CO_2_ sublimation (e.g., Diniega et al., 2010; Dundas et al., 2012; Dundas et al., 2015; Dundas et al., 2017; Pilorget & Forget, 2016; Pasquon et al., 2019). Our model results thus suggest that the fluidization obtained by CO_2_ sublimation is of the same order of the fluidization obtained by water in terrestrial granular debris flows.

Our results provide quantitative data to test and refine the qualitative model of Dundas et al. (2017) for the fluidization of the CO_2_‐triggered recent flows in Martian gullies, in which gas generation occurs via two effects within a mix of sediment and CO_2_ ice tumbling down a gully. Dundas et al. (2017) hypothesize that the potential energy of falling material is initially converted to kinetic energy but ultimately dissipates as heat or latent heat loss (sublimation) if buffered at the CO_2_ frost point temperature. Subsequently, eroded sediment from the shallow subsurface or unfrosted areas will be warmer than the ice, and could cause additional sublimation. We suggest that this sediment may also contain additional CO_2_ ice. Mixing within the falling material will allow the generated heat to be transferred to the CO_2_ frost, causing sublimation, enhancing the gas pressure within the pores of the flowing mass leading to fluidization of the flow. Below, we quantitatively test if our back‐calculated flow conditions can be explained by CO_2_ sublimation fluidizing the flow under Martian atmospheric conditions.

When a mass flow contains a volume fraction *ϕ* (‐) of CO_2_ frost, then, where bed entrainment is happening at rate $\dot{Q}$ (m/s), there will be a volume flux of CO_2_ frost $\phi \dot{Q}$ being entrained into the bottom of the flow and this then sublimates rapidly to produce a volume flux *q*_*g*_ of gaseous CO_2_ (m/s) within the flow:

(5) $$q_{g}=\frac{\rho_{i}}{\rho_{g}}\phi \dot{Q}$$

where *ρ*_*i*_ is the CO_2_ solid density (1.6 × 10^3^ kg/m^3^) and *ρ*_*g*_ is the CO_2_ gas density (1.0 × 10^−2^ kg/m^3^) at an assumed pressure of 500 Pa and a temperature of 270 K. We use assumed pressure and temperature because we did not know the exact timing of the studied flows. Assuming other reasonable values of pressure and temperature would slightly affect CO_2_ gas density and viscosity but would not affect our overall results. The large size of the $\frac{\rho_{i}}{\rho_{g}}$ ratio on Mars (1.6×10^5^) means that even a very low entrainment rate of CO_2_ ice can produce significant flow rates of gas and a substantial pore pressure. On Earth atmospheric pressure is 100–200 times larger, which increases the density of CO_2_ gas by a similar factor, yielding $\frac{\rho_{i}}{\rho_{g}}$ ≈ 800–1,600. Thus a much higher volume fraction of CO_2_ and a correspondingly higher heat flux would be necessary to fluidize a flow by CO_2_ sublimation on Earth.

Applying Darcy's law to a mass flow with a flow depth *h* (m) with permeability *k* yields a basal pore pressure:

(6) $$p=\frac{hq_{g}\nu }{k}.$$

where *ν* is the viscosity of CO_2_ gas (1.3 × 10^−5^ Pa/s). The solid normal stress at the bottom of the flow will be reduced by this pore pressure to

(7) $$N=\rho_{s}gh\cos\theta -p=\rho_{s}gh\cos\theta -\frac{h\nu \dot{Q}}{k}\frac{\rho_{i}}{\rho_{g}}\phi$$

where *ρ*_*s*_ is the sediment density (≈3,000 kg/m^3^), and *p* is the pore pressure of CO_2_ gas within the flow (Pa). The Coloumb frictional component of resistance is then reduced to

(8) $$\begin{array}{ccc} S & =\mu \left(\rho_{s}gh\cos\theta -p\right) & \\ & =\mu \left(\rho_{s}gh\cos\theta -\frac{h\nu \dot{Q}}{k}\frac{\rho_{i}}{\rho_{g}}\phi \right)\\ & =\mu \rho_{s}gh\cos\theta \left(1-\frac{\nu \dot{Q}}{k\rho_{s}g\cos\theta }\frac{\rho_{i}}{\rho_{g}}\phi \right). \end{array}$$

Thus, the reduction in friction is linear with *ϕ* and the flow will be completely fluidized when the volume fraction (‐) of solid CO_2_ is

(9) $$\phi =\frac{k\rho_{s}g\rho_{g}\cos\theta }{\nu \rho_{i}\dot{Q}}$$

The minimum required volume fraction of CO_2_ that is required to fluidize the flow is thus independent of the flow depth *h*. Although a thicker flow needs a higher pore pressure to support it, the pore pressure is proportional to flow depth because of Darcy's law.

Now consider the time scale over which the suspension can be maintained. Suppose that the erosion is rapid compared to the escape of the gas. Then since *e*_*m*_ meters of sediment is eroded, this will introduce *e*_*g*_ meters of CO_2_ gas into the flow where

(10) $$e_{g}=\phi e_{m}\frac{\rho_{i}}{\rho_{g}}.$$

The flow rate of this gas through the bulk of the material is

(11) $$\frac{k}{\nu }\frac{dp}{dz}$$

from Darcy's law. This sets the rate at which the CO_2_ gas produced by entrainment escapes so that

(12) $$\frac{de_{g}}{dt}=-\frac{k}{\nu }\frac{dp}{dz}=-\frac{k}{\nu }\frac{h\rho_{s}g\cos\theta }{h}.$$

Once again the flow depth cancels out and we have the simple solution:

(13) $$e_{g}=\phi e_{m}\frac{\rho_{i}}{\rho_{g}}-t\frac{k\rho_{s}g\cos\theta }{\nu }$$

where *t* is time (s). The flow will be suspended while the gas is still flowing, that is, while *e*_*g*_>0 so the suspension stops when *e*_*g*_(*t*)=0. Thus, the approximate time of suspension (*T* in s) is then

(14) $$T=\phi e_{m}\frac{\rho_{i}}{\rho_{g}}\frac{\nu }{k\rho_{s}g\cos\theta }.$$

This result is in contrast to usual pore pressure diffusive time scales (e.g., wet debris flows), which we would expect to scale as the flow thickness squared. This is because though a thicker flow makes it harder for the gas to escape, the driving pressure is correspondingly larger.

**Figure 13:**
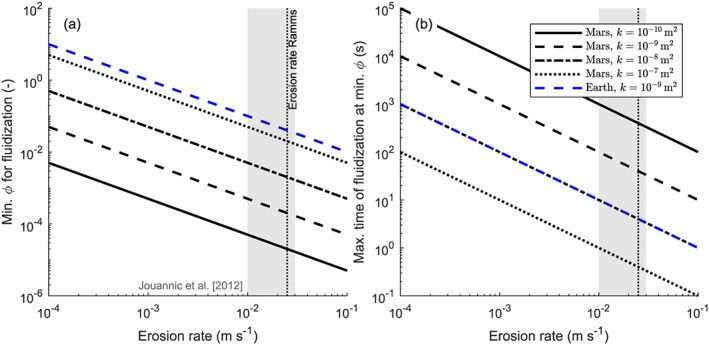
Minimum required volume fraction of CO_2_ in the sediment needed to fluidize the flow (a) and the time needed for the minimum required volume fraction of CO_2_ to escape the flow (b), as a function of sediment erosion rate. See Table 3 for input parameters. The dashed gray line indicates the erosion rate used in RAMMS, while the gray band indicates the range of erosion rates found on the Russell crater dune gullies by Jouannic et al. (2012). RAMMS = RApid Mass Movement Simulation.

These calculations show that CO_2_ sublimation is able to fluidize the studied recent flows in Hale crater (Figures 9 and 10). Assuming a channel slope of 20°, a maximum entrainment depth of 0.1 m, and a sediment density of 3,000 kg/m^3^, we find that to fluidize a flow at an entrainment rate of 0.025 m/s, as used in our RAMMS simulations and within the range of erosion rates in recent flows in gullies in the Russell crater dune (Jouannic et al., 2012), we need volume fractions of CO_2_ ranging from 2 × 10^−2^ to 2 × 10^−5^ for a permeability ranging from 10^−10^ to 10^−7^ m^2^, respectively (Figure 13a). This corresponds to a surficial frost layer of only 2 × 10^−3^ to 2 × 10^−6^ m, respectively, and even less if we assume that part of this frost is within the pores of the eroded sediment. Very low fractions of CO_2_ are thus required to fluidize the flows at these conditions. The energy necessary to sublimate such small quantities of CO_2_ necessary for fluidization will be negligible. For very small erosion rates, large volume fractions of CO_2_ are needed in relatively permeable flows to maintain fluidization. For example, for a flow with a permeability of 10^−7^ m^2^ a CO_2_ volume fraction of 0.1 or higher is needed when the erosion rate becomes smaller than 2 × 10^−2^ m/s, and an erosion rate of 5 × 10^−3^ is required for flows with a permeability of 10^−8^ m^2^—such large quantities of CO_2_ are likely not present on the surface of Martian gullies.

CO_2_ ice creates spectral signatures at near‐infrared wavelengths in CRISM (Compact Reconnaissance Imaging Spectrometer for Mars; Murchie et al., 2007) and OMEGA (Observatoire pour la Minéralogie, l'Eau, les Glaces, et l'Activité; Bibring et al., 2005) near‐infrared data that enable identification and characterization of thin ice deposits (typically few hundreds of micrometers of CO_2_ ice; Vincendon, 2015). We thus find that the CO_2_ frost thickness at the surface that is required to obtain fluidization is around the detection limit and possibly below it.

**Table 3:** Parameters for Modeling of Fluidization by CO_2_ Sublimation in Figure 13

Parameter	Value
*Variable parameters*	
Erosion rate $\dot{Q}$	10^−4^–10^−1^ m/s
Flow permiability *k*	10^−10^–10^−7^ m^2^
*Fixed parameters*	
Sediment density *ρ*_*s*_	3,000 kg/m^3^
CO_2_ solid density *ρ*_*i*_	1.6 × 10^3^ kg/m^3^
CO_2_ gas density *ρ*_*g*_	1.0 × 10^−2^ kg/m^3^
CO_2_ viscosity *η*	1.3 × 10^−5^ Pa/s
Depth of eroded CO_2_ gas *e*_*m*_	0.1 m
Channel gradient *θ*	20°

We can only guess the permeability of contemporary flows in Martian gullies. The hydraulic permeability of terrestrial debris flows typically ranges between 10^−13^ and 10^−9^ m^2^ (De Haas et al., 2015d; Iverson, 1997; Iverson et al., 2010), but the back calculated granular nature of the recent flows in Hale crater suggests that the Martian contemporary gully flows may have a relatively high permeability, although this remains speculative. In the terrestrial environment, where $\frac{\rho_{i}}{\rho_{g}}$ is much smaller, the minimum volume fraction of CO_2_ to fluidize a flow is a ∼200 larger (Figure 13a).

Figure 13b gives the time it takes for the minimum fraction of CO_2_ required to fluidize a flow, as given in Figure 13a, to fully escape the flow. This time can be interpreted as the maximum time over which fluidization can be maintained. For flows with an erosion rate of 0.025 m/s and a permeability ranging from 10^−10^ to 10^−7^ m^2^ this time ranges from 0.4 to 400 s, respectively. Given that the minimum amounts of CO_2_ required to fluidize the flow are very small for low‐permeability flows, larger amounts of CO_2_ are probably entrained. The model thus shows that it is likely for contemporary flows in Martian gullies to remain fluidized for at least tens of seconds to minutes, corresponding to the back‐calculated length of the flow events of approximately 300–1,500 s during which time bed material is entrained.

In short, our model shows that CO_2_ sublimation may indeed fluidize recent flows in Martian gullies. Although our calculations have neglected many features, including compressibility and expansion leading affecting permeability, the calculations show that even very small amounts of CO_2_ can have a strong and long‐lasting fluidization effects. The fluidization effect of CO_2_ sublimation is so strong under Martian conditions that changes in permeability by several orders of magnitude would not affect our conclusions.

### 5.2 Gravitational Effects in RAMMS

Our model is sensitive to gravity. In the Voellmy‐Salm fluid model (Salm, 1993; Voellmy, 1955) used in RAMMS, gravity cancels out in the flow equations. However, the entrainment model used in RAMMS introduces gravity dependence as follows. Basal shear stress, which determines the amount of entrainment, depends on gravity (equation (4)). Mars's gravity is about 2.5 times less than that on the Earth, and therefore basal shear stress is similarly smaller on Mars. However, if the material is noncohesive, the forces resisting entrainment will be reduced in proportion. So there is no reason to expect significant differences. If, however, there are cohesive forces resisting entrainment, then entrainment rates would be reduced on Mars. For the fixed entrainment parameters that we have applied here, we only correctly back calculate travel distance and erosion distance for gully 26, whereas we underestimate the erosion distance for gullies 9 and 69 suggesting that the erosion threshold could be lower for these gully systems.

## 6 Conclusions

We have modified the RAMMS debris flow and avalanche model to permit its use under Martian conditions, in order to back calculate and infer initial and flow conditions in three recent flows in gullies on the wall of Hale crater on Mars. These flows are generally believed to have been formed by CO_2_ sublimation, and here we constrain their initial failure conditions, flow dynamics including velocity and flow depth, and the rate of fluidization within the flows.

The studied flows are typical for recent flows in gullies on Mars. They are generated from a restricted slope failure in the gully catchment, erode bed, and bank material, mobilize boulders >0.5 m during flow, and have a final deposit with a restricted thickness of a few decimeters at maximum. We are able to accurately reproduce the observed flows, and find that the flows require a slope failure with a minimum release depth of 1.0–1.5 m, corresponding to release volumes of 100–200 m^3^. The flows grow in size by a factor ∼2.5–5.5 by entraining bed materials, and entrainment is necessary to meet the observed travel distance. Mean flow velocities in the channel generally decrease from 3–4 to ∼1 m/s, and flow depths decrease from 0.5–1 to 0.1–0.2 m, from the alcove headwaters to the flow termination point. Transition from erosion to deposition typically occurs around slopes of 13–18°, and both mean erosion depth and deposition are generally subtle, around 0.1–0.2 m.

Based on back calculation of the Voellmy‐Salm friction parameters, we show that the recent flows in Martian gullies experience similar friction as debris flows on Earth, and differ from terrestrial rock avalanches, ice‐rock avalanches, snow avalanches, and pyroclastic flows. Specifically, our model results suggest that the fluidization in contemporary flows in Martian gullies is of the same order as the fluidization in terrestrial granular debris flows. The best‐fit friction parameters are a dry‐Coulomb friction of 0.1–0.25, well below that of any natural materials, and a viscous‐turbulent friction of 100–200 m/s^2^. Through a novel model for mass flow fluidization by CO_2_ sublimation, we show that very small volumetric fractions of CO_2_ of ≪1% within mass flows may yield gas fluxes that are large enough to fluidize and enhance the mobility of recent flows in Martian gullies.

## Supporting information



Supporting Information S1

Movie S1

Movie S2

Movie S3

Movie S4

Movie S5

Movie S6

Movie S7

Movie S8

Figure S1

Figure S2

## Appendix A Vöellmy Resistance Parameter Combinations From Literature

### A.1

**Table A1:** Source Data for Figure 12

	Release							
Flow	volume		*ξ*		Back			
type	(m^3^)	*μ*	(m/s^2^)	Model	calculated	Entrained	Location	Reference
DF	1.50E+3	0.12	500	RAMMS	yes	yes	Gadria, Italy	De Finis et al. (2018)
DF	1.50E+4	0.08	300	RAMMS	yes	no	Arundakopfbach, Italy	Scheidl et al. (2013)
DF	7.00E+4	0.18	350	RAMMS	yes	no	Seefeldbach, Italy	Scheidl et al. (2013)
DF	1.00E+5	0.07	200	RAMMS	yes	no	Illgraben, Switzerland	Berger et al. (2012)
DF	1.50E+4	0.18	500	RAMMS	yes	no	Fiames 1, Italy	Cesca and d'Agostino (2008)
DF	1.06E+4	0.2	40	RAMMS	yes	no	Fiames 2, Italy	Cesca and d'Agostino (2008)
DF	4.68E+4	0.19	15	RAMMS	yes	no	Fiames 3, Italy	Cesca and d'Agostino (2008)
DF	1.10E+4	0.37	40	RAMMS	yes	no	Fiames 4, Italy	Cesca and d'Agostino (2008)
DF	5.20E+3	0.39	100	RAMMS	yes	no	Fiames 5, Italy	Cesca and d'Agostino (2008)
DF	2.10E+3	0.45	1,000	RAMMS	yes	no	Fiames 6, Italy	Cesca and d'Agostino (2008)
DF	1.00E+4	0.225	130	RAMMS	yes	no	Dorfbach, Switzerland	Deubelbeiss and Graf (2013)
DF	1.27E+2	0.4	200	RAMMS	yes	no	Napf, Switzerland	Fan et al. (2017)
DF	6.50E+4	0.3	200	RAMMS	yes	yes	Spreitgraben, Switzerland	Frank et al. (2015)
DF	1.00E+1	0.6	200	RAMMS	yes	yes	Meretschibach, Switzerland	Frank et al. (2017)
DF		0.3	400	RAMMS	yes	yes	Bondasca, Switzerland	Frank et al. (2017)
DF	1.67E+4	0.06	500	RAMMS	yes	yes	Faucon, France	Hussin et al. (2012)
DF	2.50E+4	0.11	200	RAMMS	yes	no	Reiselehnrinne, Austria	Schraml et al. (2015)
DF	2.56E+5	0.35	500	RAMMS	yes	yes	Leva Reka, Serbia	Krušić et al. (2018)
DF	1.80E+2	0.1	1,400	RAMMS	yes	no	Disentis, Switzerland	McArdell and Bartelt (2012)
DF	7.10E+1	0.4	150	RAMMS	yes	no	Alpachstad, Switzerland	Loup et al. (2012)
DF	2.90E+2	0.2	1,000	RAMMS	yes	no	Reggisberg, Switzerland	Loup et al. (2012)
DF	3.00E+1	0.1	500	RAMMS	yes	no	Kniz, Switzerland	Loup et al. (2012)
DF	2.50E+3	0.15	150	RAMMS	yes	no	Walchensee, Germany	Scheuner et al. (2011)
DF	2.25E+4	0.11	200	RAMMS	yes	no	Reiselehnrinne, Austria	Schraml et al. (2015)
DF	2.25E+4	0.08	400	DAN3D	yes	no	Reiselehnrinne, Austria	Schraml et al. (2015)
DF	1.00E+4	0.07	300	RAMMS	yes	no	Festetic, Austria	Schraml et al. (2015)
DF	1.00E+4	0.07	300	DAN3D	yes	no	Festetic, Austria	Schraml et al. (2015)
DF	1.00E+4	0.12	500	RAMMS	yes	no	Corno, Italy	Simoni et al. (2012)
DF	3.00E+3	0.27	500	RAMMS	yes	no	Campipietra, Italy	Simoni et al. (2012)
DF	3.46E+4	0.1	200	RAMMS	yes	no	Valburga, Italy	Simoni et al. (2012)
DF	9.50E+4	0.15	100	RAMMS	yes	no	Lega01, Italy	Simoni et al. (2012)
DF	3.50E+4	0.1	110	RAMMS	yes	no	Lega02, Italy	Simoni et al. (2012)
DF	1.70E+4	0.1	150	RAMMS	yes	no	Lega03, Italy	Simoni et al. (2012)
DF	3.50E+3	0.14	400	RAMMS	yes	no	Becolle, Italy	Simoni et al. (2012)
DF	5.00E+2	0.15	500	DAN3D	yes	yes	Tsing Shan, Hong Kong	Hungr (2008)
DF	9.20E+4	0.08	200	DAN‐W	yes	no	Hummingbird Creek, Canada	McKinnon et al. (2008)
DF	6.40E+3	0.06	120	‐	yes	no	Kamikamihori, Japan	Naef et al. (2006)
DF	1.75E+2	0.07	200	DAN	yes	yes	Avella, Italy	Revellino et al. (2008)
DF	9.00E+5	0.1	1,000	DAN3D	yes	yes	Zymoetz River, Canada	McDougall et al. (2006)
DA	3.10E+3	0.3	1,750	DAN3D	yes	no	DA‐1, Faroe Islands	Dahl et al. (2013)
DA	5.49E+2	0.3	1,750	DAN3D	yes	no	DA‐2, Faroe Islands	Dahl et al. (2013)
DA	6.36E+2	0.48	1,750	DAN3D	yes	no	DA‐3, Faroe Islands	Dahl et al. (2013)
DA	1.93E+3	0.19	1,750	DAN3D	yes	no	DA‐4, Faroe Islands	Dahl et al. (2013)
LL	2.82E+6	0.42	2,000	RAMMS	no	no	Xinzhuang, China	Chung et al. (2018)
LL	1.50E+6	0.18	400	RAMMS	yes	no	Chenjiaba, China	Huang et al. (2017)
LL	5.00E+6	0.13	450	DAN3D	yes	yes	Andins Valley, Switzerland	Hungr and McDougall (2009)
LL	3.60E+7	0.1	500	DAN3D	yes	no	Frank Slide, Canada	McKinnon et al. (2008)
LL	4.73E+7	0.15	500	DAN3D	yes	no	Hope Slide, Canada	McKinnon et al. (2008)
RA	3.60E+7	0.1	500	DAN3D	yes	yes	Frank Slide 1903, Canada	Hungr (2008)
RA	1.10E+7	0.19	2,100	RAMMS	yes	no	Mount Fletcher 1992, New Zealand	Allen et al. (2009)
RA	1.50E+5	0.14	3,000	RAMMS	yes	no	Vampire Peak 2008, New Zealand	Allen et al. (2009)
RA	1.50E+5	0.14	2,000	RAMMS	yes	no	Vampire Peak 2008, New Zealand	Allen et al. (2009)
RA		0.01	2,000	RAMMS	yes	no	Huascaran 1970, Peru	Allen et al. (2009)
RA		0.15	4,000	RAMMS	yes	no	Mt Cook 1991, New Zealand	Allen et al. (2009)
RA		0.2	2,000	RAMMS	yes	no	Brenva 1997, Italy	Allen et al. (2009)
RA		0.05	2,700	RAMMS	yes	no	Kolka‐Karmadon 2002, Russia	Allen et al. (2009)
RA		0.08	2,000	RAMMS	yes	no	Liamna 2003, Alaska	Allen et al. (2009)
RA	9.40E+4	0.05	400	DAN3D	yes	yes	Eagle Pass, Canada	Hungr and Evans (2004)
RA	3.75E+5	0.05	400	DAN3D	yes	yes	Nomash River, Canada	Hungr and Evans (2004)
RA	3.00E+7	0.1	500	DAN	yes	yes	Val Pola, Italy	Hungr and Evans (1996)
RA	3.00E+7	0.2	1,000	RASH3D	yes	no	Val Pola, Italy	Pirulli and Mangeney (2008)
RA	3.00E+7	0.1	700	DAN	yes	yes	Frank Slide, Canada	Hungr and Evans (1996)
RA	3.00E+7	0.1	700	RASH3D	yes	no	Frank Slide, Canada	Pirulli and Mangeney (2008)
RA	2.50E+6	0.05	1,000	DAN3D	yes	no	Thurwieser, Italy	Sosio et al. (2008)
RA	2.00E+8	0.02	250	DAN‐W	yes	no	Avalanch Lake, Canada	McKinnon et al. (2008)
RA	3.00E+6	0.125	500	DAN‐W	yes	no	Jonas Creek, Canada	McKinnon et al. (2008)
RA	9.40E+5	0.05	400	DAN‐W	yes	no	Eagle Pass, Canada	McKinnon et al. (2008)
RA	3.00E+5	0.05	400	DAN‐W	yes	no	Nomash River Slide, Canada	McKinnon et al. (2008)
RA	7.20E+5	0.1	1,000	DAN3D	yes	no	Zymoetz River, Canada	McKinnon et al. (2008)
RA	7.40E+7	0.1	500	DAN3D	yes	no	McAuley Creek, Canada	McKinnon et al. (2008)
RA	1.10E+7	0.12	400	DAN3D	yes	yes	Leyte Island, Phillippines	Evans et al. (2007)
RI	3.50E+5	0.12	1,000	RAMMS	yes	yes	Mount HualcÃa¸n, Peru	Schaub et al. (2016)
RI	6.20E+6	0.1	4,650	RAMMS	yes	yes	Iliama Red Glacier, Alaska	Schneider et al. (2012)
RI	1.18E+7	0.15	4,000	RAMMS	yes	yes	Mt Cook 1991, New Zealand	Schneider et al. (2012)
RI	8.05E+7	0.052	1,525	DAN3D	yes	yes	Mount Steele, Canada	Lipovsky et al. (2008)
SA	6.00E+4	0.19	2,100	RAMMS	no	no	Zengili, Turkey	Aydin et al. (2014)
SA	6.00E+4	0.24	1,500	RAMMS	no	no	Yaylan, Turkey	Aydin et al. (2014)
SA	3.04E+4	0.19	2,500	RAMMS	yes	no	Davraz Ski Center, Turkey	Aydin et al. (2017)
SA	1.03E+5	0.155	2,000	RAMMS	no	yes	A 816, Switzerland	Bartelt et al. (2012)
SA	1.03E+4	0.38	1,000	RAMMS	yes	no	Cerro Ventana, Argentina	Casteller et al. (2008)
SA		0.2	250	RAMMS	yes	yes	Gatschiefer, Switzerland	Christen et al. (2008)
SA	1.75E+5	0.16	2,000	RAMMS	yes	yes	In den Arelen, Switzerland	Christen et al. (2010b)
SA	5.19E+4	0.4	300	RAMMS	yes	yes	Vallée de la Sionne 5233, Switzerland	Christen et al. (2010c)
SA	7.86E+4	0.46	600	RAMMS	yes	yes	Vallée de la Sionne 506, Switzerland	Christen et al. (2010c)
SA	5.00E+3	0.55	1,500	RAMMS	no	yes	Mixed (dry‐wet avalanche); general	Dreier et al. (2014)
SA	2.00E+4	0.55	1,800	RAMMS	yes	yes	Vallée de la Sionne 628, Switzerland	Dreier et al. (2016)
SA	3.00E+4	0.55	2,000	RAMMS	yes	yes	Vallée de la Sionne 816, Switzerland	Dreier et al. (2016)
SA	5.00E+3	0.26	2,000	RAMMS	no	yes	Swiss and German Alps	Feistl et al. (2014)
SA	1.00E+4	0.32	3,0000	RAMMS	yes	yes	Vassdalen, Norway	Issler et al. (2012)
SA	1.05E+4	0.4	3,0000	RAMMS	yes	yes	Gaukheidalen, Norway	Issler et al. (2012)
SA	3.60E+4	0.22	2,200	RAMMS	yes	yes	Indre Standal, Norway	Issler et al. (2012)
SA	2.24E+2	0.6	2,000	RAMMS	yes	yes	Seehore March 2010, Italy	Maggioni et al. (2012)
SA	7.10E+2	0.5	2,000	RAMMS	yes	yes	Seehore December 2010, Italy	Maggioni et al. (2012)
SA	2.04E+2	0.6	2,000	RAMMS	yes	yes	Seehore 5 march 2011, Italy	Maggioni et al. (2012)
SA	1.32E+3	0.5	2,000	RAMMS	yes	yes	Seehore 19 march 2011, Italy	Maggioni et al. (2012)
SA	9.38E+4	0.4	1,300	RAMMS	yes	yes	Gatschiefer, Switzerland	Vera Valero et al. (2015)
SA	6.49E+4	0.4	1,300	RAMMS	yes	yes	Salezer, Switzerland	Vera Valero et al. (2015)
SA	2.42E+2	0.55	1,300	RAMMS	yes	yes	Andina, Chile	Vera Valero et al. (2015)
SA	6.96E+3	0.5	1,300	RAMMS	yes	yes	Bird Hill, Alaska	Vera Valero et al. (2015)
SA		0.13	3,500	VARA	yes	no	Isafjrdur, Iceland	Barbolini et al. (2000)
SA		0.15	3,500	SFISAR	yes	no	Isafjrdur, Iceland	Barbolini et al. (2000)
SA		0.15	3,500	MN2D	yes	no	Isafjrdur, Iceland	Barbolini et al. (2000)
SA		0.35	4,500	VARA	yes	no	Mettlenruns, Switzerland	Barbolini et al. (2000)
SA		0.35	1,500	SFISAR	yes	no	Mettlenruns, Switzerland	Barbolini et al. (2000)
SA		0.3	1,000	MN2D	yes	no	Mettlenruns, Switzerland	Barbolini et al. (2000)
SA		0.35	2,000	VARA	yes	no	Pastuira, Spain	Barbolini et al. (2000)
SA		0.35	1,500	SFISAR	yes	no	Pastuira, Spain	Barbolini et al. (2000)
SA		0.4	1,000	MN2D	yes	no	Pastuira, Spain	Barbolini et al. (2000)
SA		0.18	3,500	VARA	yes	no	Ribal, France	Barbolini et al. (2000)
SA		0.2	1,000	SFISAR	yes	no	Ribal, France	Barbolini et al. (2000)
SA		0.22	1,000	MN2D	yes	no	Ribal, France	Barbolini et al. (2000)
SA		0.2	3,500	VARA	yes	no	Voltage, Italy	Barbolini et al. (2000)
SA		0.28	1,500	SFISAR	yes	no	Voltage, Italy	Barbolini et al. (2000)
SA		0.3	1,000	MN2D	yes	no	Voltage, Italy	Barbolini et al. (2000)
PF	4.20E+4	0.19	1,000	DAN3D	yes	no	Stomboli volcano, Italy	Salvatici et al. (2016)

*Note.* DF = Debris Flow; DA = Debris Avalanche; LS = Landslide; RA = Rock avalanche; RI = Rock‐ice avalanche; SA = Snow avalanche; PF = Pyroclastic Flow.

## References

[jgre21192-bib-0001] Allen, S. , Schneider, D. , & Owens, I. (2009). First approaches towards modelling glacial hazards in the Mount Cook region of New Zealand's Southern Alps. Natural Hazards and Earth System Sciences, 9(2), 481–499.

[jgre21192-bib-0002] Atwood‐Stone, C. , & McEwen, A. S. (2013). Avalanche slope angles in low‐gravity environments from active Martian sand dunes. Geophysical Research Letters, 40, 2929–2934. 10.1002/grl.50586

[jgre21192-bib-0003] Auld, K. S. , & Dixon, J. C. (2016). A classification of Martian gullies from HiRISE imagery. Planetary and Space Science, 131, 88–101.

[jgre21192-bib-0004] Aydin, A. , Bühler, Y. , Christen, M. , & Gürer, I. (2014). Avalanche situation in Turkey and back calculation of selected events. Natural Hazards and Earth System Sciences, 14(5), 1145–1154.

[jgre21192-bib-0005] Aydin, A. , Eker, R. , & Ersan, H. (2017). Back‐calculation Of 8 January 2012 snow avalanche in Davraz Ski Center in International Symposium on New Horizons in Forestry.

[jgre21192-bib-0006] Balmforth, N. J. , & McElwaine, J. N. (2018). From episodic avalanching to continuous flow in a granular drum. Granular Matter, 20(3), 52.

[jgre21192-bib-0007] Barbolini, M. , Gruber, U. , Keylock, C. , Naaim, M. , & Savi, F. (2000). Application of statistical and hydraulic‐continuum dense‐snow avalanche models to five real European sites. Cold Regions Science and Technology, 31(2), 133–149. 10.1016/S0165-232X(00)00008-2

[jgre21192-bib-0008] Bartelt, P. , Bieler, C. , Buehler, Y. , Christen, M. , Deubelbeiss, Y. , Graf, C. , McArdell, B. , Salz, M. , & Schneider, M. (2017). RAMMS::DEBRISFLOW user manual, WSL.

[jgre21192-bib-0009] Bartelt, P. , Böhler, Y. , Buser, O. , Christen, M. , & Meier, L. (2012). Modeling mass‐dependent flow regime transitions to predict the stopping and depositional behavior of snow avalanches. Journal of Geophysical Research, 117, F01015 10.1029/2010JF001957

[jgre21192-bib-0010] Berger, C. , McArdell, B. W. , Fritschi, B. , & Schlunegger, F. (2010). A novel method for measuring the timing of bed erosion during debris flows and floods. Water Resources Research, 46, W02502 10.1029/2009WR007993

[jgre21192-bib-0011] Berger, C. , McArdell, B. W. , & Lauber, G. (2012). Debris flow simulation at Illgraben, Switzerland, using 2D numerical model RAMMS in 12th Congress Interpraevent.

[jgre21192-bib-0012] Berger, C. , McArdell, B. , & Schlunegger, F. (2011). Direct measurement of channel erosion by debris flows, Illgraben, Switzerland. Journal of Geophysical Research 2003–2012, 116, F01002 10.1029/2010JF001722

[jgre21192-bib-0013] Bibring, J.‐P. , Langevin, Y. , Gendrin, A. , Gondet, B. , Poulet, F. , Berthé, M. , Soufflot, A. , Arvidson, R. , Mangold, N. , Mustard, J. , Drossart, P. , OMEGA team (2005). Mars surface diversity as revealed by the OMEGA/Mars Express observations. Science, 307, 1576–1581. 15718430 10.1126/science.1108806

[jgre21192-bib-0014] Carling, P. , & Glaister, M. (1987). Rapid deposition of sand and gravel mixtures downstream of a negative step: The role of matrix‐infilling and particle‐overpassing in the process of bar‐front accretion. Journal of the Geological Society, 144(4), 543–551.

[jgre21192-bib-0015] Casteller, A. , Christen, M. , Villalba, R. , Martínez, H. , Stöckli, V. , Leiva, J. C. , & Bartelt, P. (2008). Validating numerical simulations of snow avalanches using dendrochronology: The Cerro Ventana event in Northern Patagonia, Argentina. Natural Hazards and Earth System Sciences, 8(3), 433–443. 10.5194/nhess-8-433-2008

[jgre21192-bib-0016] Cedillo‐Flores, Y. , Treiman, A. H. , Lasue, J. , & Clifford, S. M. (2011). CO_2_ gas fluidization in the initiation and formation of Martian polar gullies. Geophysical Research Letters, 38, L21202 10.1029/2011GL049403

[jgre21192-bib-0017] Cesca, M. , & d'Agostino, V. (2008). Comparison between FLO‐2D and RAMMS in debris‐flow modelling: A case study in the Dolomites. WIT Transactions on Engineering Sciences, 60, 197–206.

[jgre21192-bib-0018] Christen, M. , Bartelt, P. , & Kowalski, J. (2010b). Back calculation of the in Den Arelen avalanche with RAMMS: Interpretation of model results. Annals of Glaciology, 51(54), 161–168.

[jgre21192-bib-0019] Christen, M. , Bartelt, P. , Kowalski, J. , & Stoffel, L. (2008). Calculation of dense snow avalanches in three‐dimensional terrain with the numerical simulation program RAMMS. Proceedings Whistler 2008 International Snow Science Workshop September, 21–27.

[jgre21192-bib-0020] Christen, M. , Bühler, Y. , Bartelt, P. , Leine, R. , Glover, J. , Schweizer, A. , Graf, C. , McArdell, B. W. , Gerber, W. , Deubelbeiss, Y. , Feistl, T. , & Volkwein, A. (2012). Integral hazard management using a unified software environment. In *12th Congress Interpraevent* (pp. 77–86). Grenoble, France: Interpraevent.

[jgre21192-bib-0021] Christen, M. , Kowalski, J. , & Bartelt, P. (2010a). RAMMS: Numerical simulation of dense snow avalanches in three‐dimensional terrain. Cold Regions Science and Technology, 63(1‐2), 1–14.

[jgre21192-bib-0022] Christen, M. , Kowalski, J. , & Bartelt, P. (2010c). Ramms: Numerical simulation of dense snow avalanches in three‐dimensional terrain. Cold Regions Science and Technology, 63(1), 1–14. 10.1016/j.coldregions.2010.04.005

[jgre21192-bib-0023] Chung, M.‐C. , Chen, C.‐H. , Lee, C.‐F. , Huang, W.‐K. , & Tan, C.‐H. (2018). Failure impact assessment for large‐scale landslides located near human settlement: Case study in southern Taiwan. Sustainability, 10(5), 1–25.30607262

[jgre21192-bib-0024] Conway, S. J. , & Balme, M. R. (2014). Decameter thick remnant glacial ice deposits on Mars. Geophysical Research Letters, 41, 5402–5409. 10.1002/2014GL060314

[jgre21192-bib-0025] Conway, S. J. , & Balme, M. (2016). A novel topographic parameterization scheme indicates that Martian gullies display the signature of liquid water. Earth and Planetary Science Letters, 454, 36–45.

[jgre21192-bib-0026] Conway, S. J. , Balme, M. R. , Kreslavsky, M. A. , Murray, J. B. , & Towner, M. C. (2015). The comparison of topographic long profiles of gullies on Earth to gullies on Mars: A signal of water on Mars. Icarus, 253, 189–204.

[jgre21192-bib-0027] Conway, S. J. , Balme, M. R. , Murray, J. B. , Towner, M. C. , Okubo, C. H. , & Grindrod, P. M. (2011). The indication of Martian gully formation processes by slope–area analysis. Geological Society, London, Special Publications, 356(1), 171–201.

[jgre21192-bib-0028] Conway, S. J. , Butcher, F. E. , de Haas, T. , Deijns, A. J. , Grindrod, P. M. , & Davis, J. M. (2018a). Glacial and gully erosion on Mars: A terrestrial perspective. Geomorphology, 318, 26–57.

[jgre21192-bib-0029] Conway, S. J. , de Haas, T. , & Harrison, T. (2018b). Martian gullies: A comprehensive review of observations, mechanisms and insights from Earth analogues. Geological Society, London, Special Publications, 467, SP467–14.

[jgre21192-bib-0030] Conway, S. , Decaulne, A. , Balme, M. , Murray, J. , & Towner, M. (2010). A new approach to estimating hazard posed by debris flows in the Westfjords of Iceland. Geomorphology, 114(4), 556–572.

[jgre21192-bib-0031] Conway, S. , Harrison, T. , Soare, R. , Britton, A. , & Steele, L. (2017). New slope‐normalized global gully density and orientation maps for Mars. Geological Society, London, Special Publications, 467, SP467–3.

[jgre21192-bib-0032] Costard, F. , Forget, F. , Mangold, N. , & Peulvast, J. P. (2002). Formation of recent Martian debris flows by melting of near‐surface ground ice at high obliquity. Science, 295, 110–113. 11729267 10.1126/science.1066698

[jgre21192-bib-0033] Dahl, M.‐P. J. , Gauer, P. , Kalsnes, B. G. , Mortensen, L. E. , Jensen, N. H. , & Veihe, A. (2013). Numerical runout simulation of debris avalanches in the Faroe Islands, North Atlantic Ocean. Landslides, 10(5), 623–631.

[jgre21192-bib-0034] De Finis, E. , Gattinoni, P. , Marchi, L. , & Scesi, L. (2018). Anomalous Alpine fans: From the genesis to the present hazard. Landslides, 15(4), 683–694.

[jgre21192-bib-0035] De Haas, T. , Braat, L. , Leuven, J. F. W. , Lokhorst, I. R. , & Kleinhans, M. G. (2015d). Effects of debris‐flow composition and topography on runout distance, depositional mechanisms and deposit morphology. Journal of Geophysical Research: Earth Surface, 120, 1949–1972. 10.1002/2015JF003525

[jgre21192-bib-0036] De Haas, T. , Conway, S. , Butcher, F. , Levy, J. , Grindrod, P. , Goudge, T. , & Balme, M. (2017). Time will tell: Temporal evolution of Martian gullies and palaeoclimatic implications. Geological Society, London, Special Publications, 467.

[jgre21192-bib-0037] De Haas, T. , Conway, S. J. , & Krautblatter, M. (2015a). Recent (Late Amazonian) enhanced backweathering rates on Mars: Paracratering evidence from gully alcoves. Journal of Geophysical Research: Planets, 120, 2169–2189. 10.1002/2015JE004915

[jgre21192-bib-0038] De Haas, T. , Hauber, E. , Conway, S. J. , van Steijn, H. , Johnsson, A. , & Kleinhans, M. G. (2015b). Earth‐like aqueous debris‐flow activity on Mars at high orbital obliquity in the last million years. Nature Communications, 6, 7543. 10.1038/ncomms8543 PMC4557294 26102485

[jgre21192-bib-0039] De Haas, T. , & Van Woerkom, T. (2016). Bed scour by debris flows: Experimental investigation of effects of debris‐flow composition. Earth Surface Processes and Landforms, 41(13), 1951–1966.

[jgre21192-bib-0040] De Haas, T. , Ventra, D. , Hauber, E. , Conway, S. J. , & Kleinhans, M. G. (2015c). Sedimentological analyses of Martian gullies: the subsurface as the key to the surface. Icarus, 258, 92–108.

[jgre21192-bib-0041] Deubelbeiss, Y. , & Graf, C. (2013). Two different starting conditions in numerical debris flow models—Case study at Dorfbach, Randa (Valais, Switzerland), GRAF, C.(Red.) Mattertal–ein Tal in Bewegung. Publikation zur Jahrestagung der Schweizerischen Geomorphologischen Gesellschaft, 29, 125–138.

[jgre21192-bib-0042] Dickson, J. L. , Head, J. W. , & Kreslavsky, M. (2007). Martian gullies in the southern mid‐latitudes of Mars: Evidence for climate‐controlled formation of young fluvial features based upon local and global topography. Icarus, 188(2), 315–323.

[jgre21192-bib-0043] Diniega, S. , Byrne, S. , Bridges, N. T. , Dundas, C. M. , & McEwen, A. S. (2010). Seasonality of present‐day Martian dune‐gully activity. Geology, 38(11), 1047–1050.

[jgre21192-bib-0044] Dreier, L. , Böhler, Y. , Ginzler, C. , & Bartelt, P. (2016). Comparison of simulated powder snow avalanches with photogrammetric measurements. Annals of Glaciology, 57(71), 371–381. 10.3189/2016AoG71A532

[jgre21192-bib-0045] Dreier, L. , Böhler, Y. , Steinkogler, W. , Feistl, T. , Christen, M. , & Bartelt, P. (2014). Modelling small and frequent snow avalances. *Proceedings, International Snow Science Workshop, Banff, 2014* (pp. 649–656).

[jgre21192-bib-0046] Dundas, C. M. , Diniega, S. , Hansen, C. J. , Byrne, S. , & McEwen, A. S. (2012). Seasonal activity and morphological changes in Martian gullies. Icarus, 220(1), 124–143.

[jgre21192-bib-0047] Dundas, C. M. , Diniega, S. , & McEwen, A. S. (2015). Long‐term monitoring of Martian gully formation and evolution with MRO/HiRISE. Icarus, 251, 244–263.

[jgre21192-bib-0048] Dundas, C. M. , McEwen, A. S. , Diniega, S. , Byrne, S. , & Martinez‐Alonso, S. (2010). New and recent gully activity on Mars as seen by HiRISE. Geophysical Research Letters, 37, L07202 10.1029/2009GL041351

[jgre21192-bib-0049] Dundas, C. M. , McEwen, A. S. , Diniega, S. , Hansen, C. , Byrne, S. , & McElwaine, J. N. (2017). The formation of gullies on Mars today. Geological Society, London, Special Publications, 467, SP467–5.

[jgre21192-bib-0050] Evans, S. , Guthrie, R. , Roberts, N. , & Bishop, N. (2007). The disastrous 17 February 2006 rockslide‐debris avalanche on Leyte Island, Philippines: A catastrophic landslide in tropical mountain terrain. Natural Hazards and Earth System Science, 7(1), 89–101.

[jgre21192-bib-0051] Fan, L. , Lehmann, P. , McArdell, B. , & Or, D. (2017). Linking rainfall‐induced landslides with debris flows runout patterns towards catchment scale hazard assessment. Geomorphology, 280, 1–15.

[jgre21192-bib-0052] Feistl, T. , Bebi, P. , Teich, M. , Böhler, Y. , Christen, M. , Thuro, K. , & Bartelt, P. (2014). Observations and modeling of the braking effect of forests on small and medium avalanches. Journal of Glaciology, 60(219), 124–138. 10.3189/2014JoG13J055

[jgre21192-bib-0053] Fischer, J.‐T. , Kowalski, J. , & Pudasaini, S. P. (2012). Topographic curvature effects in applied avalanche modeling. Cold Regions Science and Technology, 74, 21–30.

[jgre21192-bib-0054] Frank, F. , McArdell, B. , Huggel, C. , & Vieli, A. (2015). The importance of entrainment and bulking on debris flow runout modeling: Examples from the Swiss Alps. Natural Hazards and Earth System Sciences, 15(11), 2569.

[jgre21192-bib-0055] Frank, F. , McArdell, B. W. , Oggier, N. , Baer, P. , Christen, M. , & Vieli, A. (2017). Debris‐flow modeling at Meretschibach and Bondasca catchments, Switzerland: sensitivity testing of field‐data‐based entrainment model. Natural Hazards and Earth System Sciences,17(5), 801.

[jgre21192-bib-0056] Gulick, V. C. , Glines, N. , Hart, S. , & Freeman, P. (2019). Geomorphological analysis of gullies on the central peak of Lyot Crater, Mars. Geological Society, London, Special Publications, 467(1), 233–265.

[jgre21192-bib-0057] Guthrie, R. , Hockin, A. , Colquhoun, L. , Nagy, T. , Evans, S. , & Ayles, C. (2010). An examination of controls on debris flow mobility: Evidence from coastal British Columbia. Geomorphology, 114(4), 601–613.

[jgre21192-bib-0058] Harrison, T. N. (2016). Martian gully formation and evolution: Studies from the local to global scale.

[jgre21192-bib-0059] Harrison, T. N. , Osinski, G. R. , Tornabene, L. L. , & Jones, E. (2015). Global documentation of gullies with the Mars Reconnaissance Orbiter Context Camera and implications for their formation. Icarus, 252, 236–254.

[jgre21192-bib-0060] Hartmann, W. K. , Thorsteinsson, T. , & Sigurdsson, F. (2003). Martian hillside gullies and Icelandic analogs. Icarus, 162(2), 259–277.

[jgre21192-bib-0061] Head, J. W. , Mustard, J. F. , Kreslavsky, M. A. , Milliken, R. E. , & Marchant, D. R. (2003). Recent ice ages on Mars. Nature, 426, 797–802. 14685228 10.1038/nature02114

[jgre21192-bib-0062] Huang, T. , Ding, M.‐T. , She, T. , Tian, S.‐J. , & Yang, J.‐T. (2017). Numerical simulation of a high‐speed landslide in Chenjiaba, Beichuan, China. Journal of Mountain Science, 14(11), 2137–2149.

[jgre21192-bib-0063] Hugenholtz, C. H. (2008). Frosted granular flow: A new hypothesis for mass wasting in Martian gullies. Icarus, 197(1), 65–72.

[jgre21192-bib-0064] Hungr, O. (2008). Numerical modelling of the dynamics of debris flows and rock avalanches. Geomechanik und Tunnelbau: Geomechanik und Tunnelbau, 1(2), 112–119.

[jgre21192-bib-0065] Hungr, O. , & Evans, S. (1996). Rock avalanche runout prediction using a dynamic model. In *Inproceedings of the 7th International Symposium on Landslides*, 11,Trondheim, Norway, pp. 233–238.

[jgre21192-bib-0066] Hungr, O. , & Evans, S. (2004). Entrainment of debris in rock avalanches: An analysis of a long run‐out mechanism. Geological Society of America Bulletin, 116(9‐10), 1240–1252.

[jgre21192-bib-0067] Hungr, O. , & McDougall, S. (2009). Two numerical models for landslide dynamic analysis. Computers & Geosciences, 35(5), 978–992.

[jgre21192-bib-0068] Hungr, O. , McDougall, S. , & Bovis, M. J. (2005). Entrainment of material by debris flows In Jakob M. & Hungr O. (Eds.), Hazard assessment of debris flows and related phenomena (Chap. 7).Heidelberg: Springer.

[jgre21192-bib-0069] Hungr, O. , Morgan, G. , & Kellerhals, R. (1984). Quantitative analysis of debris torrent hazards for design of remedial measures. Canadian Geotechnical Journal, 21(4), 663–677.

[jgre21192-bib-0070] Hussin, H. , Luna, B. Q. , Van Westen, C. , Christen, M. , Malet, J.‐P. , & van Asch, T. W. (2012). Parameterization of a numerical 2‐D debris flow model with entrainment: a case study of the Faucon catchment, Southern French Alps. Natural hazards and earth system sciences, 12(10), 3075.

[jgre21192-bib-0071] Issler, D. , Harbitz, C. B. , Domaas, U. , Kronholm, K. , & Christen, M. (2012). Back‐calculations of observed avalanches against natural deflecting d, Proceedings 2012 International Snow Science Workshop. Alaska:Anchorage.

[jgre21192-bib-0072] Iverson, R. M. (1997). The physics of debris flows. Reviews of Geophysics, 35(3), 245–296.

[jgre21192-bib-0073] Iverson, R. M. , Logan, M. , LaHusen, R. G. , & Berti, M. (2010). The perfect debris flow? Aggregated results from 28 large‐scale experiments. Journal of Geophysical Research, 115, F03005 10.1029/2009JF001514

[jgre21192-bib-0074] Iverson, R. M. , Reid, M. E. , Logan, M. , LaHusen, R. G. , Godt, J. W. , & Griswold, J. P. (2011). Positive feedback and momentum growth during debris‐flow entrainment of wet bed sediment. Nature Geoscience, 4(2), 116–121.

[jgre21192-bib-0075] Jawin, E. R. , Head, J. W. , & Marchant, D. R. (2018). Transient post‐glacial processes on Mars: Geomorphologic Evidence for a Paraglacial Period. Icarus, 309, 187–206.

[jgre21192-bib-0076] Johnsson, A. , Reiss, D. , Hauber, E. , Hiesinger, H. , & Zanetti, M. (2014). Evidence for very recent melt‐water and debris flow activity in gullies in a young mid‐latitude crater on Mars. Icarus, 235, 37–54.

[jgre21192-bib-0077] Jones, A. , McEwen, A. , Tornabene, L. , Baker, V. , Melosh, H. , & Berman, D. (2011). A geomorphic analysis of Hale crater, Mars: The effects of impact into ice‐rich crust. Icarus, 211(1), 259–272.

[jgre21192-bib-0078] Jouannic, G. , Gargani, J. , Costard, F. , Ori, G. G. , Marmo, C. , Schmidt, F. , & Lucas, A. (2012). Morphological and mechanical characterization of gullies in a periglacial environment: The case of the Russell crater dune (Mars). Planetary and Space Science, 71(1), 38–54.

[jgre21192-bib-0079] Kirk, R. , Howington‐Kraus, E. , Rosiek, M. , Anderson, J. , Archinal, B. , Becker, K. , Cook, D. , Galuszka, D. , Geissler, P. , Hare, T. , Holmberg, I. M. , Keszthelyi, L. P. , Redding, B. L. , Delamere, W. A. , Gallagher, D. , Chapel, J. D. , Eliason, E. M. , King, R. , & McEwen, A. S. (2008). Ultrahigh resolution topographic mapping of Mars with MRO HiRISE stereo images: Meter‐scale slopes of candidate Phoenix landing sites. Journal of Geophysical Research, 113, E00A24 10.1029/2007JE003000

[jgre21192-bib-0080] Kleinhans, M. , Markies, H. , De Vet, S. , & Postema, F. (2011). Static and dynamic angles of repose in loose granular materials under reduced gravity. Journal of Geophysical Research, 116, E11004 10.1029/2011JE003865

[jgre21192-bib-0081] Kolb, K. J. , McEwen, A. S. , & Pelletier, J. D. (2010). Investigating gully flow emplacement mechanisms using apex slopes. Icarus, 208(1), 132–142.

[jgre21192-bib-0082] Krušić, J. , SamardžIć‐Petrovć, M. , Marjanović, M. , Abolmasov, B. , & Miljkoviś S. (2018). Preliminary results of numerical modelling of debris flow‐case study Leva reka, Serbia. ce/papers, 2(2‐3), 707–712.

[jgre21192-bib-0083] Lamb, M. P. , Dietrich, W. E. , & Venditti, J. G. (2008). Is the critical shields stress for incipient sediment motion dependent on channel‐bed slope? Journal of Geophysical Research, 113, F02008 10.1029/2007JF000831

[jgre21192-bib-0084] Lanza, N. , Meyer, G. , Okubo, C. , Newsom, H. , & Wiens, R. (2010). Evidence for debris flow gully formation initiated by shallow subsurface water on Mars. Icarus, 205(1), 103–112.

[jgre21192-bib-0085] Levy, J. , Head, J. , Dickson, J. , Fassett, C. , Morgan, G. , & Schon, S. (2010). Identification of gully debris flow deposits in Protonilus Mensae, Mars: Characterization of a water‐bearing, energetic gully‐forming process. Earth and Planetary Science Letters, 294, 368–377.

[jgre21192-bib-0086] Lipovsky, P. S. , Evans, S. G. , Clague, J. J. , Hopkinson, C. , Couture, R. , Bobrowsky, R. , Ekström, G. , Demuth, M. N. , Delaney, K. B. , Roberts, N. J. , Clarke, G. , & Schaeffer, A. (2008). The July 2007 rock and ice avalanches at Mount Steele, St. Elias Mountains, Yukon, Canada. Landslides, 5(4), 445–455. 10.1007/s10346-008-0133-4

[jgre21192-bib-0087] Loup, B. , Egli, T. , Stucki, M. , Bartelt, P. , McArdell, B. W. , & Baumann, R. (2012). (2012), Impact pressures of hillslope debris flows in 12th Congress Interpraevent.

[jgre21192-bib-0088] Maggioni, M. , Freppaz, M. , Christen, M. , Bartelt, P. , & Zanini, E. (2012). Back‐calculation of small avalanches with the 2D avalanche dynamics model RAMMS: Four events artificially triggered at the Seehore test site in Aosta Valley (NW Italy), Proceedings 2012 International Snow Science Workshop. Alaska:Anchorage.

[jgre21192-bib-0089] Malin, M. C. , & Edgett, K. S. (2000). Evidence for recent groundwater seepage and surface runoff on Mars. Science, 288, 2330–2335. 10875910 10.1126/science.288.5475.2330

[jgre21192-bib-0090] Malin, M. C. , Edgett, K. S. , Posiolova, L. V. , McColley, S. M. , & Dobrea, E. Z. N. (2006). Present‐day impact cratering rate and contemporary gully activity on Mars. Science, 314, 1573–1577. 17158321 10.1126/science.1135156

[jgre21192-bib-0091] Mangold, N. , Costard, F. , & Forget, F. (2003). Debris flows over sand dunes on Mars: Evidence for liquid water. Journal of Geophysical Research, 108(E4), 5027 10.1029/2002JE001958

[jgre21192-bib-0092] Mangold, N. , Mangeney, A. , Migeon, V. , Ansan, V. , Lucas, A. , Baratoux, D. , & Bouchut, F. (2010). Sinuous gullies on Mars: Frequency, distribution, and implications for flow properties. Journal of Geophysical Research, 115, E11001 10.1029/2009JE003540

[jgre21192-bib-0093] McArdell, B. W. , & Bartelt, P. (2012). Open‐slope debris flows: Recent advances in runout modeling In C. K. Lau, E. Chan, & J. Kwan (Eds.), Proceedings of the one day seminar on natural terrain hazard mitigation measures, pp. 56–60.

[jgre21192-bib-0094] McDougall, S. , Boultbee, N. , Hungr, O. , Stead, D. , & Schwab, J. W. (2006). The Zymoetz River landslide, British Columbia, Canada: Description and dynamic analysis of a rock slide–debris flow. Landslides, 3(3), 195 10.1007/s10346-006-0042-3

[jgre21192-bib-0095] McEwen, A. S. , Eliason, E. M. , Bergstrom, J. W. , Bridges, N. T. , Hansen, C. J. , Delamere, W. A. , Grant, J. A. , Gulick, V. C. , Herkenhoff, K. E. , Keszthelyi, L. , Kirk, R. L. , Mellon, M. T. , Squyres, S. W. , Thomas, N. , & Weitz, C. M. (2007). Mars reconnaissance orbiter's high resolution imaging science experiment (HiRISE). Journal of Geophysical Research, 112, E05S02 10.1029/2005JE002605

[jgre21192-bib-0096] McKinnon, M. , Hungr, O. , & McDougall, S. (2008). Dynamic analyses of Canadian landslides. In *proceedings of the Fourth Canadian Conference on GeoHazards: From Causes to Management* (pp. 20–24).Laval, Québec: Presse de l'Université de Laval.

[jgre21192-bib-0097] Melosh, H. J. (1989). Impact cratering: A geologic process, Research supported by NASA. New York, Oxford University Press (Oxford Monographs on Geology and Geophysics,. No. 11, 1989, 253 p.11.

[jgre21192-bib-0098] Mergili, M. , Jan‐Thomas, F. , Krenn, J. , & Pudasaini, S. P. (2017). r. avaflow v1, an advanced open‐source computational framework for the propagation and interaction of two‐phase mass flows. Geoscientific Model Development, 10(2), 553–569.

[jgre21192-bib-0099] Milliken, R. , Mustard, J. , & Goldsby, D. (2003). Viscous flow features on the surface of Mars: Observations from high‐resolution Mars Orbiter Camera (MOC) images. Journal of Geophysical Research, 108(E6), 5057 10.1029/2002JE002005

[jgre21192-bib-0100] Murchie, S. , Arvidson, R. , Bedini, P. , Beisser, K. , Bibring, J.‐P. , Bishop, J. , Boldt, J. , Cavender, P. , Choo, T. , Clancy, R. , Darlington, E. H. , Des Marais, D. , Espiritu, R. , Fort, D. , Green, R. , Guinness, E. , Hayes, J. , Hash, C. , Heffernan, K. , Hemmler, J. , Heyler, G. , Humm, D. , Hutcheson, J. , Izenberg, N. , Lee, R. , Lees, J. , Lohr, D. , Malaret, E. , Martin, T. , McGovern, J. A. , McGuire, P. , Morris, R. , Mustard, J. , Pelkey, S. , Rhodes, E. , Robinson, M. , Roush, T. , Schaefer, E. , Seagrave, G. , Seelos, F. , Silverglate, P. , Slavney, S. , Smith, M. , Shyong, W. J. , Strohbehn, K. , Taylor, H. , Thompson, P. , Tossman, B. , Wirzburger, M. , & Wolff, M. (2007). Compact reconnaissance imaging spectrometer for Mars (CRISM) on Mars reconnaissance orbiter (MRO). Journal of Geophysical Research, 112, E05S03 10.1029/2006JE002682

[jgre21192-bib-0101] Mustard, J. F. , Cooper, C. D. , & Rifkin, M. K. (2001). Evidence for recent climate change on Mars from the identification of youthful near‐surface ground ice. Nature, 412, 411–414. 11473309 10.1038/35086515

[jgre21192-bib-0102] Naef, D. , Rickenmann, D. , Rutschmann, P. , & McArdell, B. (2006). Comparison of flow resistance relations for debris flows using a one‐dimensional finite element simulation model. Natural Hazards and Earth System Science, 6(1), 155–165.

[jgre21192-bib-0103] Okuda, S. , & Suwa, H. (1984). Some relationships between debris flow motion and micro‐topography for the Kamikamihori fan, North Japan Alps. (pp 447–464). Norwich England:Catchment Experiments in Fluvial Geomorphology. Geo Books.

[jgre21192-bib-0104] Pasquon, K. , Gargani, J. , Nachon, M. , Conway, S. J. , Massé, M. , Jouannic, G. , Balme, M. R. , Costard, F. , & Vincendon, M. (2019). Are different Martian gully morphologies due to different processes on the Kaiser dune field? Geological Society, London, Special Publications, 467(1), 145–164.

[jgre21192-bib-0105] Pelletier, J. D. , Kolb, K. J. , McEwen, A. S. , & Kirk, R. L. (2008). Recent bright gully deposits on Mars: Wet or dry flow? Geology, 36(3), 211–214.

[jgre21192-bib-0106] Pérez, F. L. (2001). Matrix granulometry of catastrophic debris flows (December 1999) in central coastal Venezuela. Catena, 45(3), 163–183.

[jgre21192-bib-0107] Pilorget, C. , & Forget, F. (2016). Formation of gullies on Mars by debris flows triggered by CO_2_ sublimation. Nature Geoscience, 9(1), 65–69.

[jgre21192-bib-0108] Pirulli, M. , & Mangeney, A. (2008). Results of back‐analysis of the propagation of rock avalanches as a function of the assumed rheology. Rock Mechanics and Rock Engineering, 41(1), 59–84.

[jgre21192-bib-0109] Raack, J. , Reiss, D. , Appéré, T. , Vincendon, M. , Ruesch, O. , & Hiesinger, H. (2015). Present‐day seasonal gully activity in a south polar pit (Sisyphi Cavi) on Mars. Icarus, 251, 226–243.

[jgre21192-bib-0110] Reid, M. E. , Iverson, R. M. , Logan, M. , LaHusen, R. G. , Godt, J. , & Griswold, J. (2011). Entrainment of bed sediment by debris flows: Results from large‐scale experiments. Debris‐flow Hazards Mitigation, Mechanics, Prediction, and Assessment, 367–374.

[jgre21192-bib-0111] Reiss, D. , Erkeling, G. , Bauch, K. , & Hiesinger, H. (2010). Evidence for present day gully activity on the Russell crater dune field, Mars. Geophysical Research Letters, 37, L06203 10.1029/2009GL042192

[jgre21192-bib-0112] Reiss, D. , Hauber, E. , Hiesinger, H. , Jaumann, R. , Trauthan, F. , Preusker, F. , Zanetti, M. , Ulrich, M. , Johnsson, A. , Johansson, L. , Olvmo, M. , Carlsson, E. , Johansson, H. A. B. , & McDaniel, S. (2011). Terrestrial gullies and debris‐flow tracks on Svalbard as planetary analogs for Mars. Geological Society of America Special Papers, 483, 165–175.

[jgre21192-bib-0113] Reiss, D. , Hiesinger, H. , Hauber, E. , & Gwinner, K. (2009). Regional differences in gully occurrence on Mars: A comparison between the Hale and Bond craters. Planetary and Space Science, 57(8‐9), 958–974.

[jgre21192-bib-0114] Reiss, D. , van Gasselt, S. , Neukum, G. , & Jaumann, R. (2004). Absolute dune ages and implications for the time of formation of gullies in Nirgal Vallis, Mars. Journal of Geophysical Research, 109, E06007 10.1029/2004JE002251

[jgre21192-bib-0115] Revellino, P. , Guadagno, F. M. , & Hungr, O. (2008). Morphological methods and dynamic modelling in landslide hazard assessment of the Campania Apennine carbonate slope. Landslides, 5(1), 59–70.

[jgre21192-bib-0116] Salm, B. (1993). Flow, flow transition and runout distances of flowing avalanches. Annals of Glaciology, 18, 221–226.

[jgre21192-bib-0117] Salvatici, T. , Roberto, A. D. , Traglia, F. D. , Bisson, M. , Morelli, S. , Fidolini, F. , Bertagnini, A. , Pompilio, M. , Hungr, O. , & Casagli, N. (2016). From hot rocks to glowing avalanches: Numerical modelling of gravity‐induced pyroclastic density currents and hazard maps at the stromboli volcano (italy). Geomorphology, 273, 93–106. 10.1016/j.geomorph.2016.08.011

[jgre21192-bib-0118] Schaub, Y. , Huggel, C. , & Cochachin, A. (2016). Ice‐avalanche scenario elaboration and uncertainty propagation in numerical simulation of rock‐/ice‐avalanche‐induced impact waves at Mount Hualcán and Lake 513, Peru. Landslides, 13(6), 1445–1459.

[jgre21192-bib-0119] Scheidl, C. , Rickenmann, D. , & McArdell, B. W. (2013). Runout prediction of debris flows and similar mass movements In Margottini C., Canuti P., & Sassa K. (Eds.), Landslide Science and Practice.Berlin, Heidelberg: Springer.

[jgre21192-bib-0120] Scheuner, T. , Schwab, S. , & McArdell, B. (2011). Application of a two‐dimensional numerical model in risk and hazard assessment in Switzerland, 5th DFHM, Padua, Italy.

[jgre21192-bib-0121] Schneider, D. , Bartelt, P. , Caplan‐Auerbach, J. , Christen, M. , Huggel, C. , & McArdell, B. W. (2010). Insights into rock‐ice avalanche dynamics by combined analysis of seismic recordings and a numerical avalanche model. Journal of Geophysical Research, 115, F04026 10.1029/2010JF001734

[jgre21192-bib-0122] Schneider, D. , Bartelt, P. , Caplan‐Auerbach, J. , Christen, M. , Huggel, C. , & McArdell, B. W. (2012). Insights into rock‐ice avalanche dynamics by combined analysis of seismic recordings and a numerical avalanche model. Journal of Geophysical Research, 115, F04026 10.1029/2010JF001734

[jgre21192-bib-0123] Schon, S. C. , Head, J. W. , & Fassett, C. I. (2009). Unique chronostratigraphic marker in depositional fan stratigraphy on Mars: Evidence for ca. 1.25 Ma gully activity and surficial meltwater origin. Geology, 37, 207–210.

[jgre21192-bib-0124] Schraml, K. , Thomschitz, B. , McArdell, B. , Graf, C. , & Kaitna, R. (2015). Modeling debris‐flow runout patterns on two alpine fans with different dynamic simulation models. Natural Hazards and Earth System Sciences, 15(7), 1483–1492.

[jgre21192-bib-0125] Schürch, P. , Densmore, A. L. , Rosser, N. J. , & McArdell, B. W. (2011). Dynamic controls on erosion and deposition on debris‐flow fans. Geology, 39(9), 827–830.

[jgre21192-bib-0126] Simoni, A. , Mammoliti, M. , & Graf, C. (2012). Performance Of 2D debris flow simulation model RAMMS. In *Annual International Conference on Geological and Earth Sciences GEOS* Singapore.

[jgre21192-bib-0127] Sosio, R. , Crosta, G. B. , & Hungr, O. (2008). Complete dynamic modeling calibration for the Thurwieser rock avalanche (Italian Central Alps). Engineering Geology, 100(1‐2), 11–26.

[jgre21192-bib-0128] Stock, J. , & Dietrich, W. E. (2003). Valley incision by debris flows: Evidence of a topographic signature. Water Resources Research, 39(4), 1089 10.1029/2001WR001057

[jgre21192-bib-0129] Sylvest, M. E. , Conway, S. J. , Patel, M. R. , Dixon, J. C. , & Barnes, A. (2016). Mass wasting triggered by seasonal CO_2_ sublimation under Martian atmospheric conditions: Laboratory experiments. Geophysical Research Letters, 43, 12,363–12,370. 10.1002/2016GL071022

[jgre21192-bib-0130] Sylvest, M. E. , Dixon, J. C. , Conway, S. J. , Patel, M. , McElwaine, J. , Hagermann, A. , & Barnes, A. (2018). CO_2_ sublimation in Martian gullies: laboratory experiments at varied slope angle and regolith grain sizes. Geological Society, London, Special Publications, 467, SP467–11.

[jgre21192-bib-0131] Takahashi, T. (2009). A review of Japanese debris flow research. International Journal of Erosion Control Engineering, 2(1), 1–14.

[jgre21192-bib-0132] Theule, J. , Liébault, F. , Laigle, D. , Loye, A. , & Jaboyedoff, M. (2015). Channel scour and fill by debris flows and bedload transport. Geomorphology, 243, 92–105.

[jgre21192-bib-0133] Treiman, A. H. (2003). Geologic settings of Martian gullies: Implications for their origins. Journal of Geophysical Research, 108(E4), 8031 10.1029/2002JE001900

[jgre21192-bib-0134] Vera Valero, C. , Jones, K. W. , Bh¨ler, Y. , & Bartelt, P. (2015). Release temperature, snow‐cover entrainment and the thermal flow regime of snow avalanches. Journal of Glaciology, 61(225), 173–184. 10.3189/2015JoG14J117

[jgre21192-bib-0135] Vincendon, M. (2015). Identification of Mars gully activity types associated with ice composition. Journal of Geophysical Research: Planets, 120, 1859–1879. 10.1002/2015JE004909

[jgre21192-bib-0136] Voellmy, A. (1955). Uber die zerstorungskraft von lawinen. Schweiz Bauztg, 73(12), 159–165.

[jgre21192-bib-0137] Vollmer, S. , & Kleinhans, M. G. (2007). Predicting incipient motion, including the effect of turbulent pressure fluctuations in the bed. Water Resources Research, 43, W05410 10.1029/2006WR004919

