# Supplementary material for: Initiation and Flow Conditions of Contemporary Flows in Martian Gullies
Source: J Geophys Res Planets. 2019 Aug 28;124(8):2246–71. doi: 10.1029/2018JE005899 (PMC6853261; doi:10.1029/2018JE005899)
Supplement: Supplementary file 1 — Supporting Information S1 [file JGRE-124-2246-s011.docx]

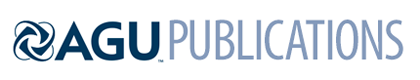


*[Journal Name]*

Supporting Information for

**Initiation and flow conditions of contemporary flows in Martian gullies**

T. de Haas^1,2^, B. W. McArdell^3^, S. J. Conway^4^, J. N. McElwaine^5,6^, M. G. Kleinhans^1^, F.

Salese^1,7^, P. M. Grindrod^8^

^1^Department of Physical Geography, Universiteit Utrecht, Utrecht, The Netherlands.

^2^Department of Geography, Durham University, Durham, UK.

^3^Swiss Federal Institute for Forest, Snow and Landscape Research WSL, Birmensdorf, Switzerland.

^4^Laboratoire de Planétologie et Géodynamique, CNRS UMR 6112, Université de Nantes, Nantes, France.

^5^Department of Earth Sciences, Durham University, Durham, UK.

^6^Planetary Science Institute, Tucson, USA.

^7^International Research School of Planetary Sciences, Universit Gabriele D’Annunzio, Pescara, Italy.

^8^Department of Earth Sciences, Natural History Museum, London, UK

**Contents of this file**

Figures S1 to S2

Movies S1 to S8

**Introduction**

The supplementary data contain two additional figures, highlighting the location of active flows in the study area (Fig S1) and highlighting the boulder mobility in the three gully systems that were modeled (Fig S2).

Furthermore, the supplementary data contains GIF movies showing before and after images of the release areas, channel changes and the terminal deposition areas of the modeled gully systems.


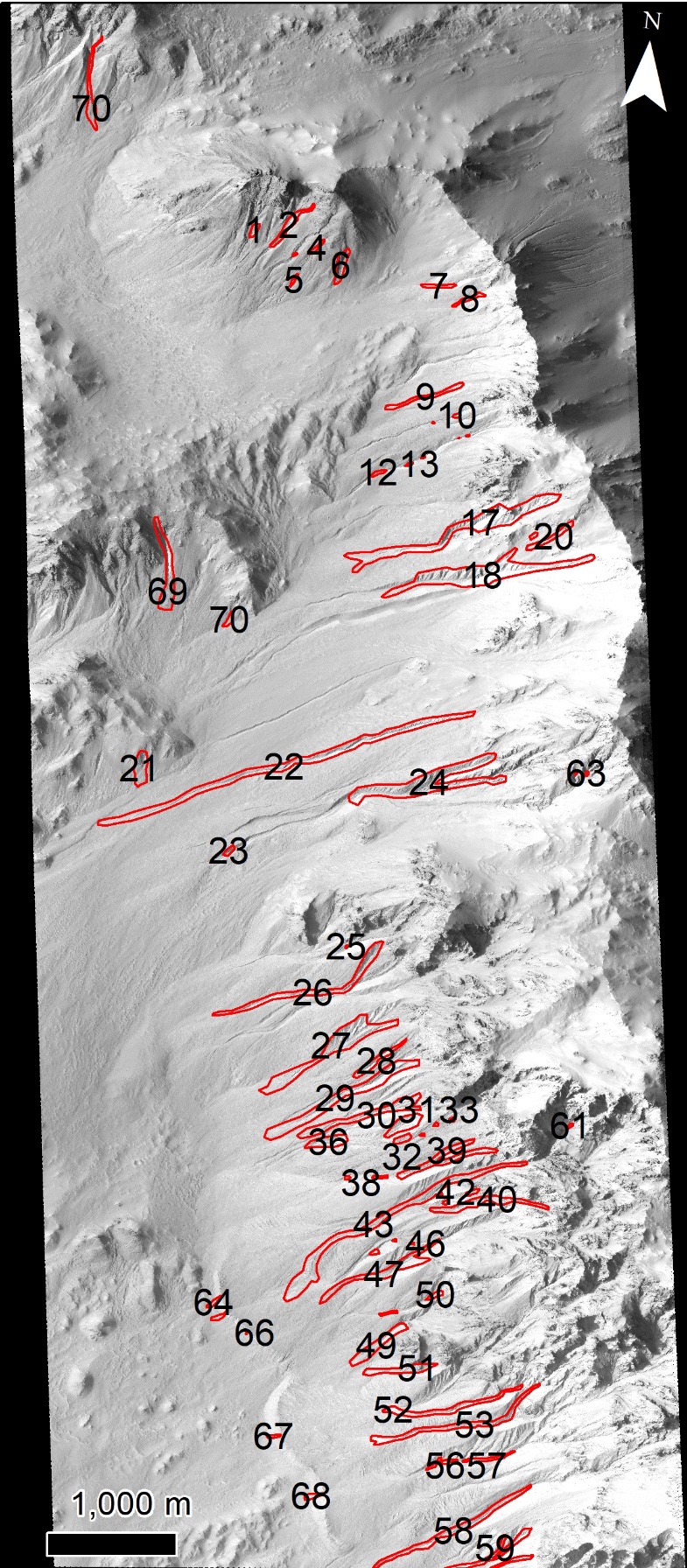


Figure S1. Locations where gully activity took place between 12 March 2007 and 21 September 2014.


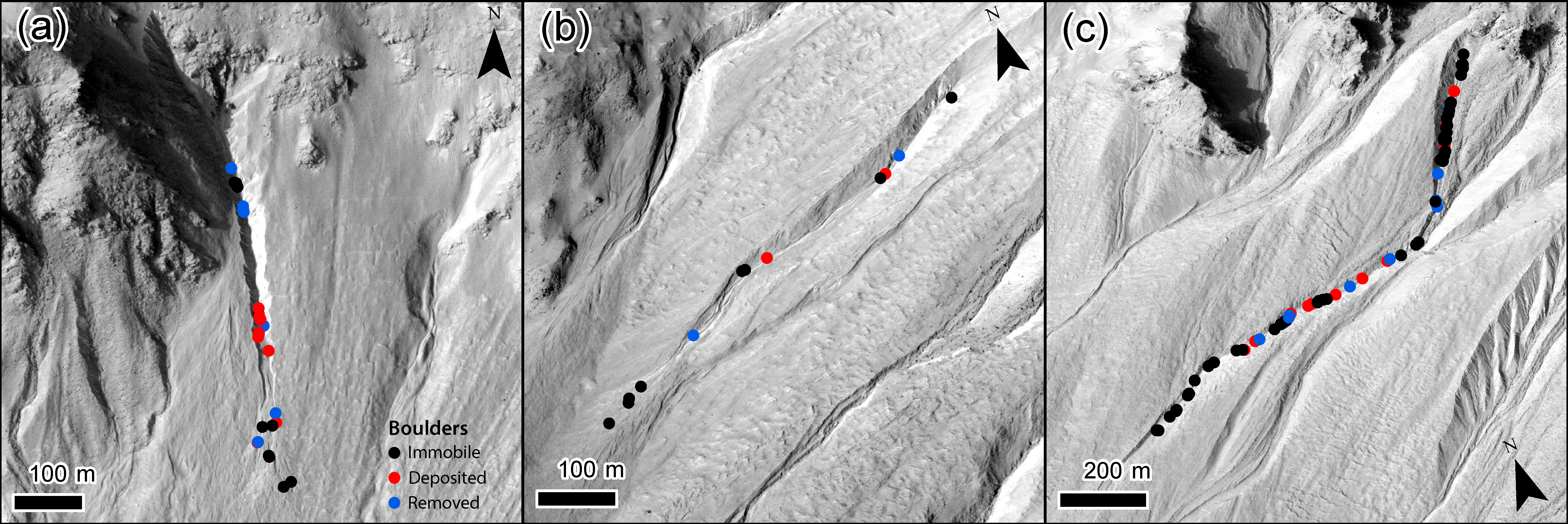


Figure S2. Boulder mobility in the three modelled gullies, as shown in Figure 3-65 and 11. (a) Gully 69. (b) Gully 9. (c) Gully 26. Gully numbers correspond to Figure S1.

Movie S1. Example of channel changes in gully 69.

Movie S2. Changes in the terminal deposition area of gully 69.

Movie S3. Changes in the terminal release area of gully 9.

Movie S4. Example of channel changes in gully 9.

Movie S5. Changes in the terminal deposition area of gully 9.

Movie S6. Changes in the terminal release area of gully 26.

Movie S7. Example of channel changes in gully 26.

Movie S8. Changes in the terminal deposition area of gully 26.
