# Supplementary figures and images for: Initiation and Flow Conditions of Contemporary Flows in Martian Gullies
Source: J Geophys Res Planets. 2019 Aug 28;124(8):2246–71. doi: 10.1029/2018JE005899 (PMC6853261; doi:10.1029/2018JE005899)

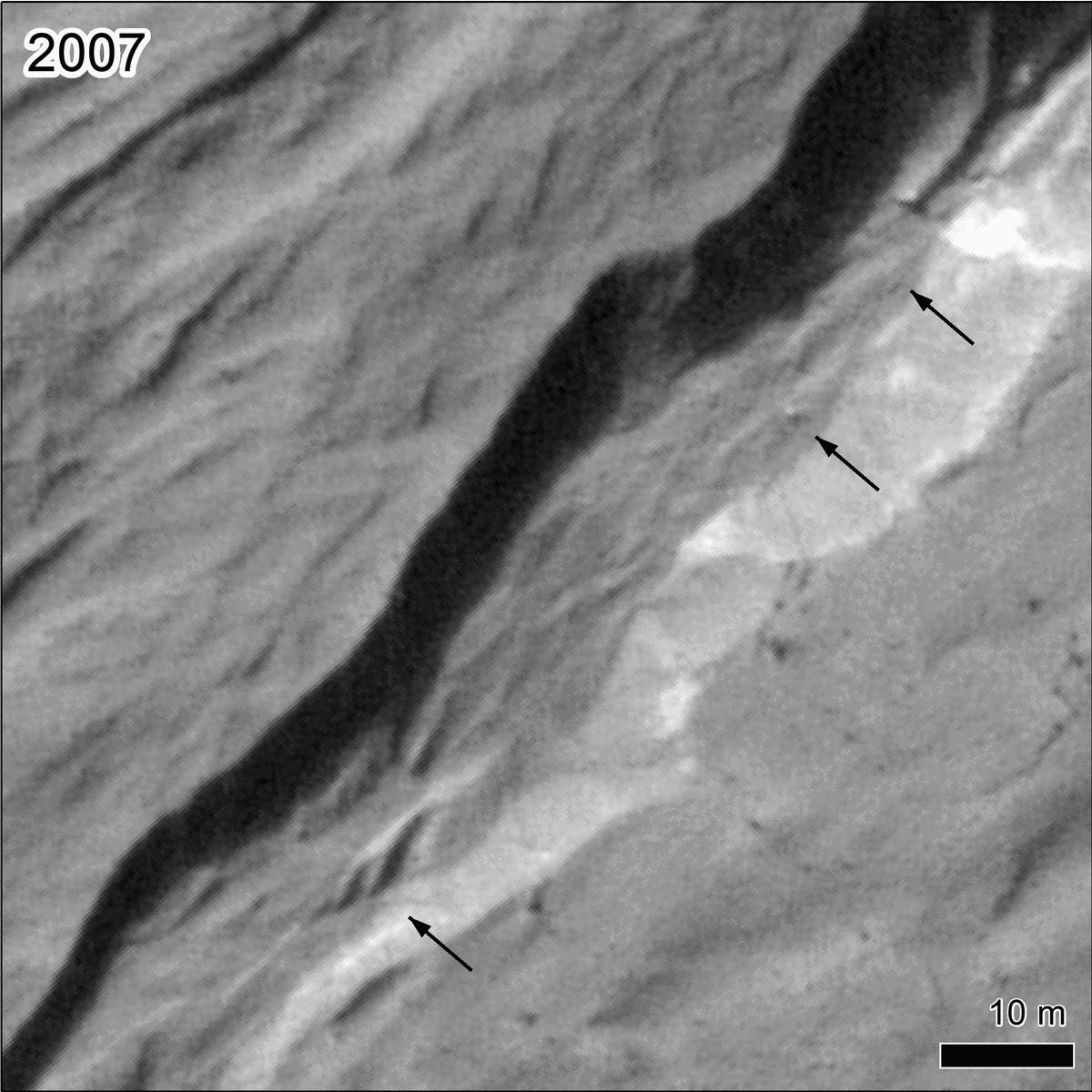

Supplement: Supplementary file 2 — Movie S1 [file JGRE-124-2246-s003.gif]

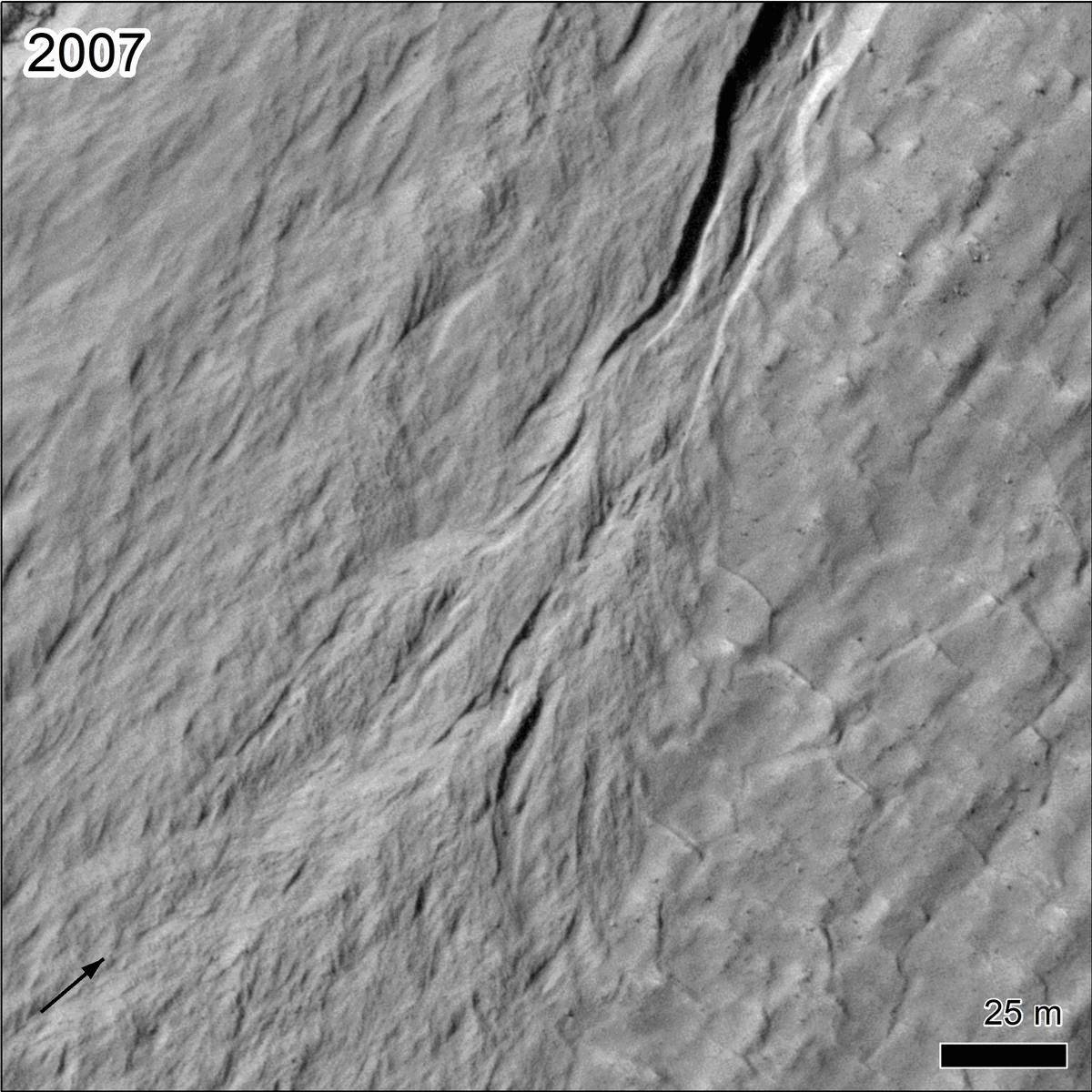

Supplement: Supplementary file 3 — Movie S2 [file JGRE-124-2246-s004.gif]

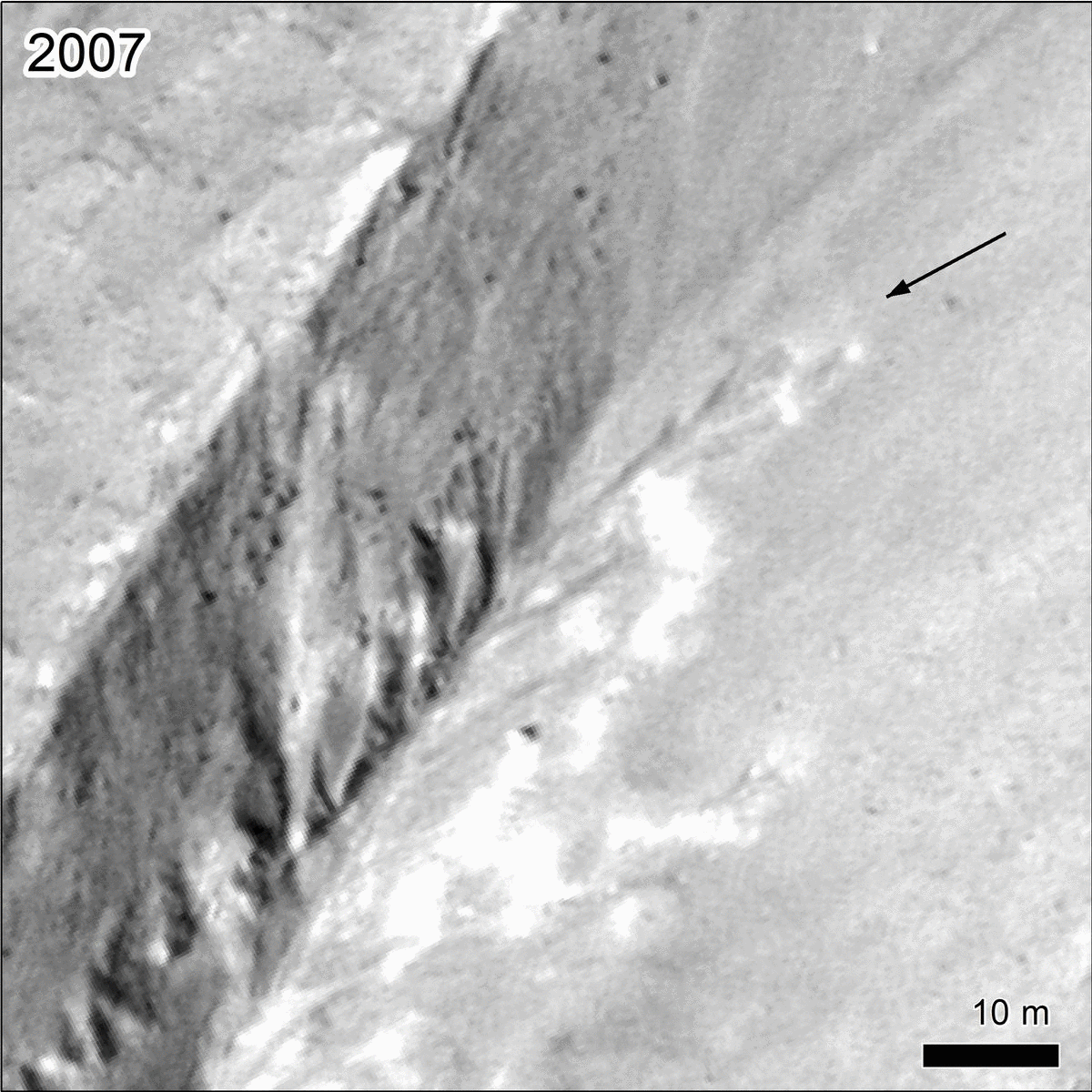

Supplement: Supplementary file 4 — Movie S3 [file JGRE-124-2246-s005.gif]

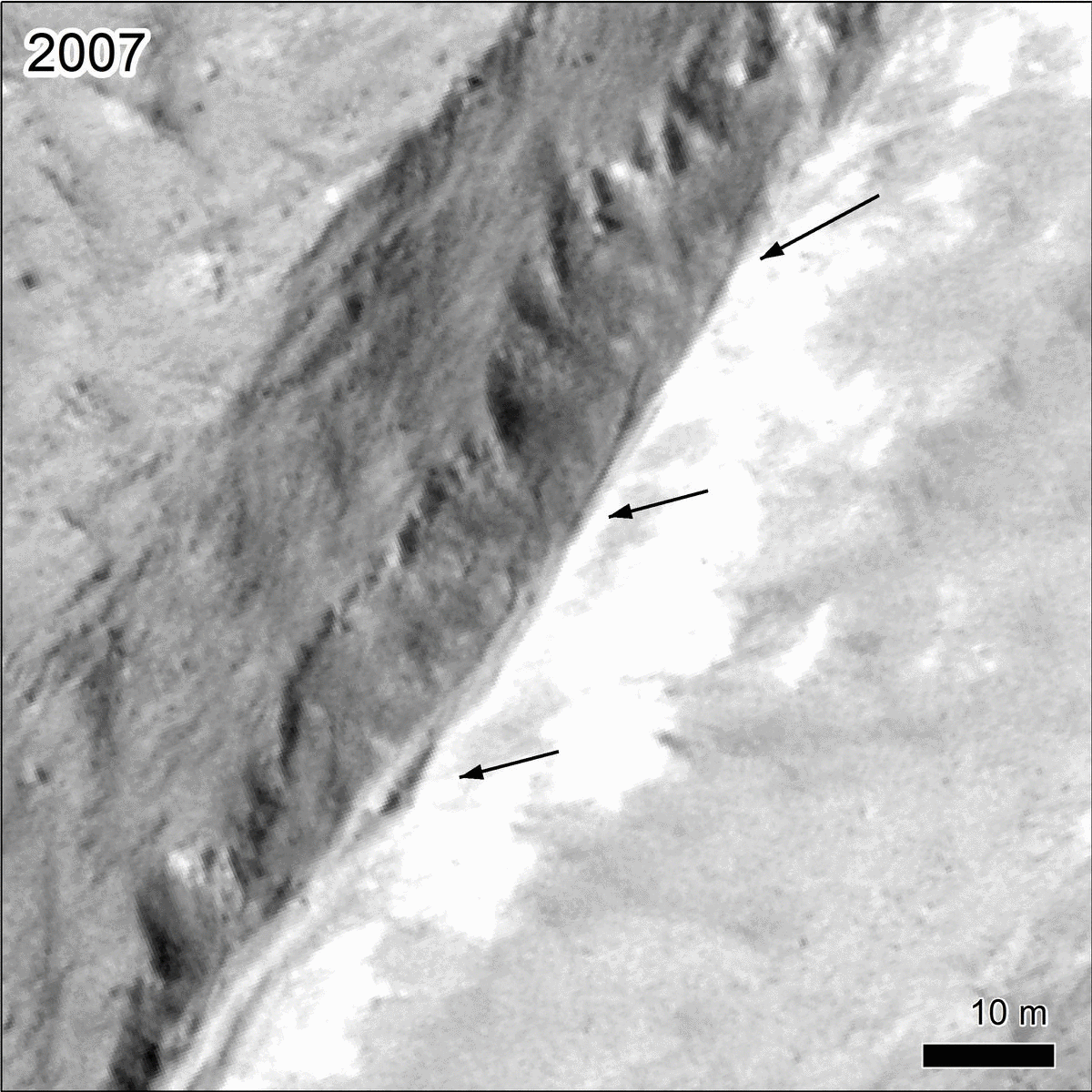

Supplement: Supplementary file 5 — Movie S4 [file JGRE-124-2246-s006.gif]

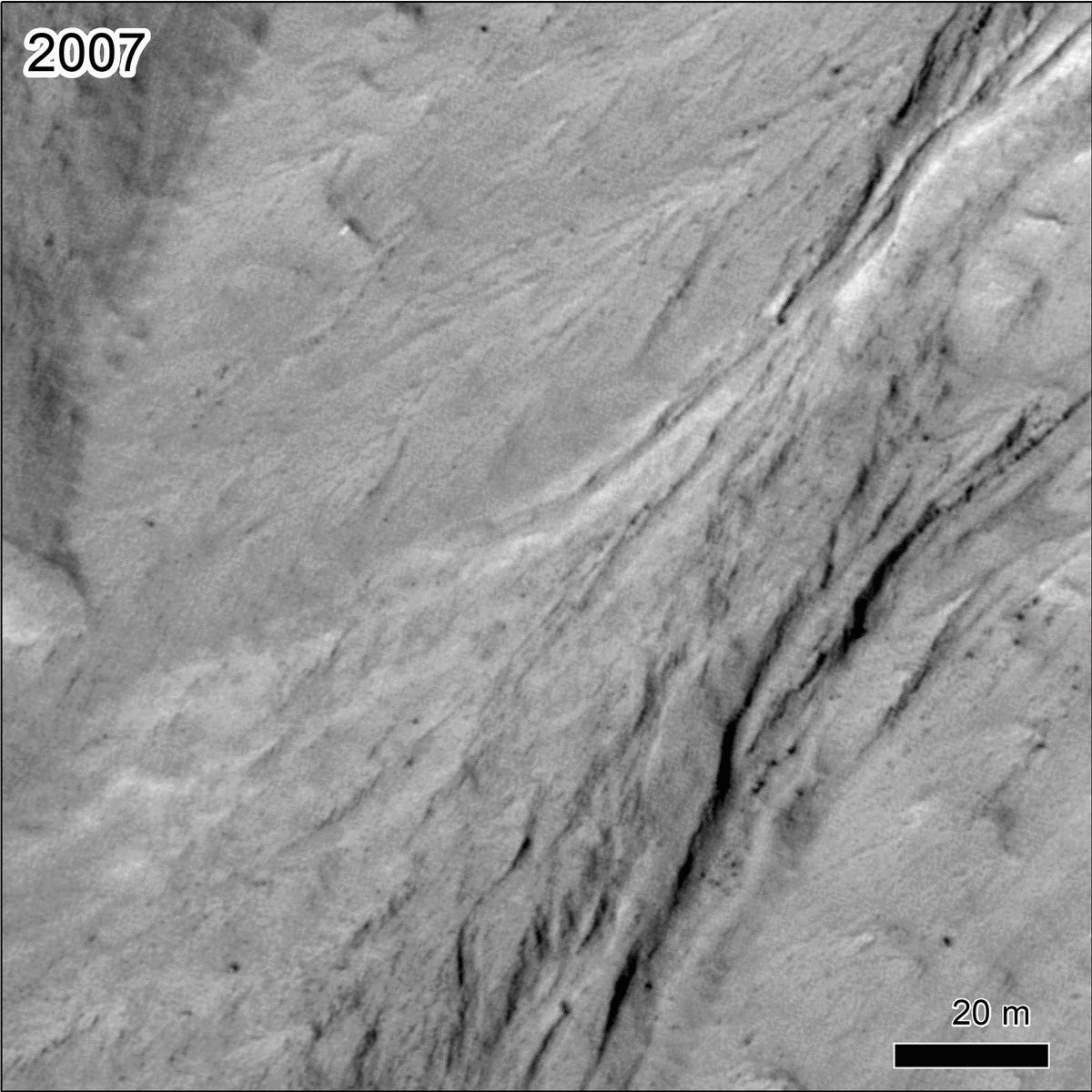

Supplement: Supplementary file 6 — Movie S5 [file JGRE-124-2246-s007.gif]

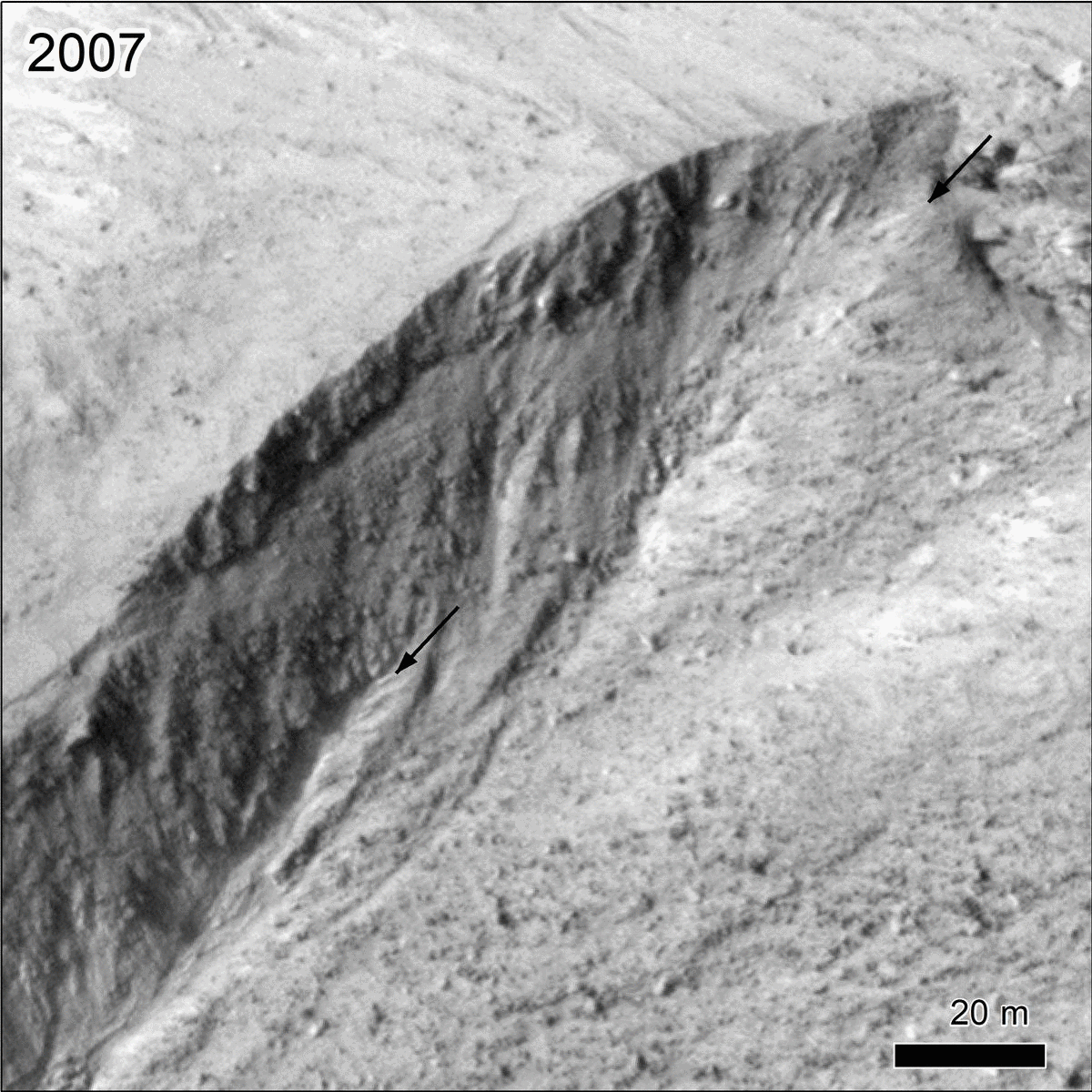

Supplement: Supplementary file 7 — Movie S6 [file JGRE-124-2246-s008.gif]

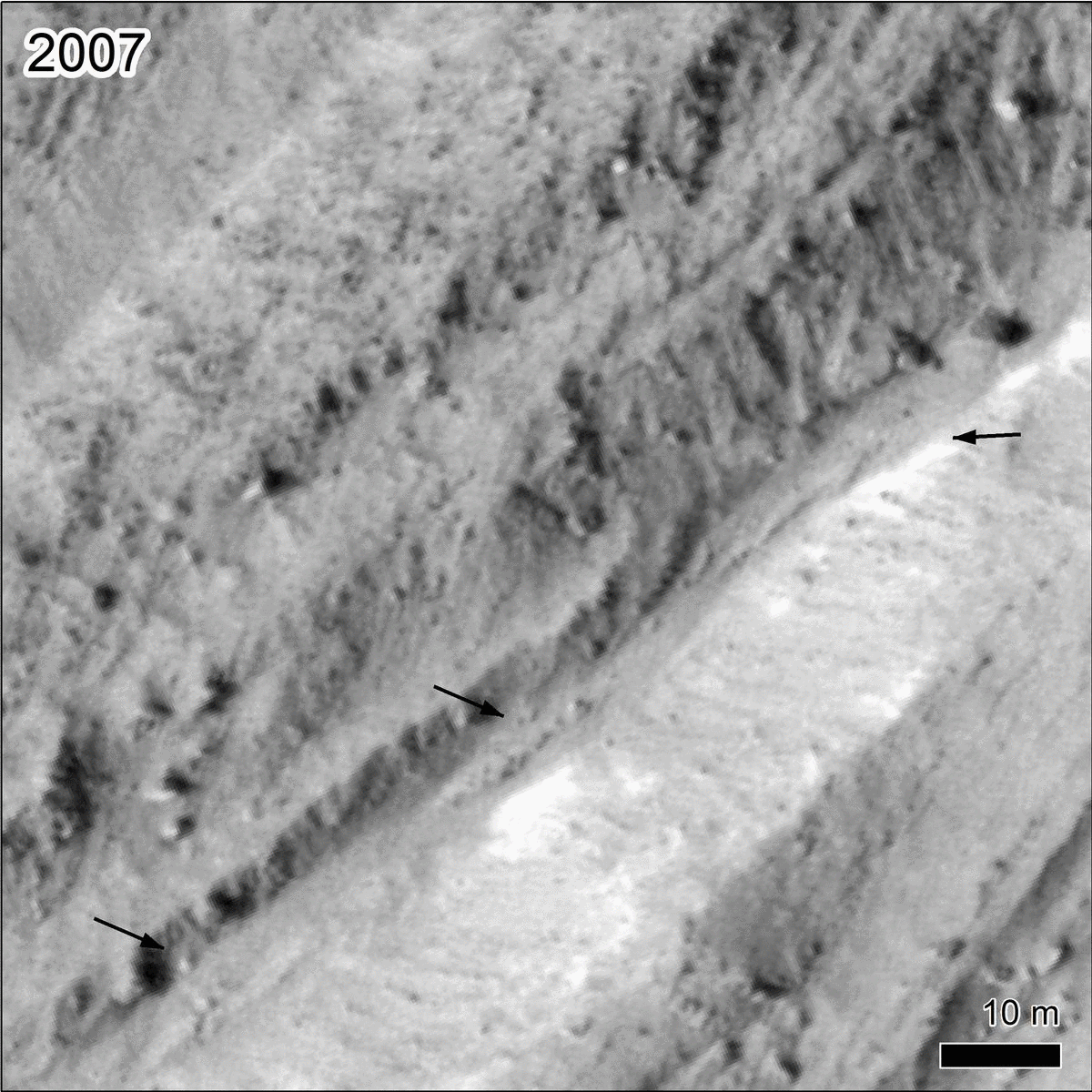

Supplement: Supplementary file 8 — Movie S7 [file JGRE-124-2246-s009.gif]

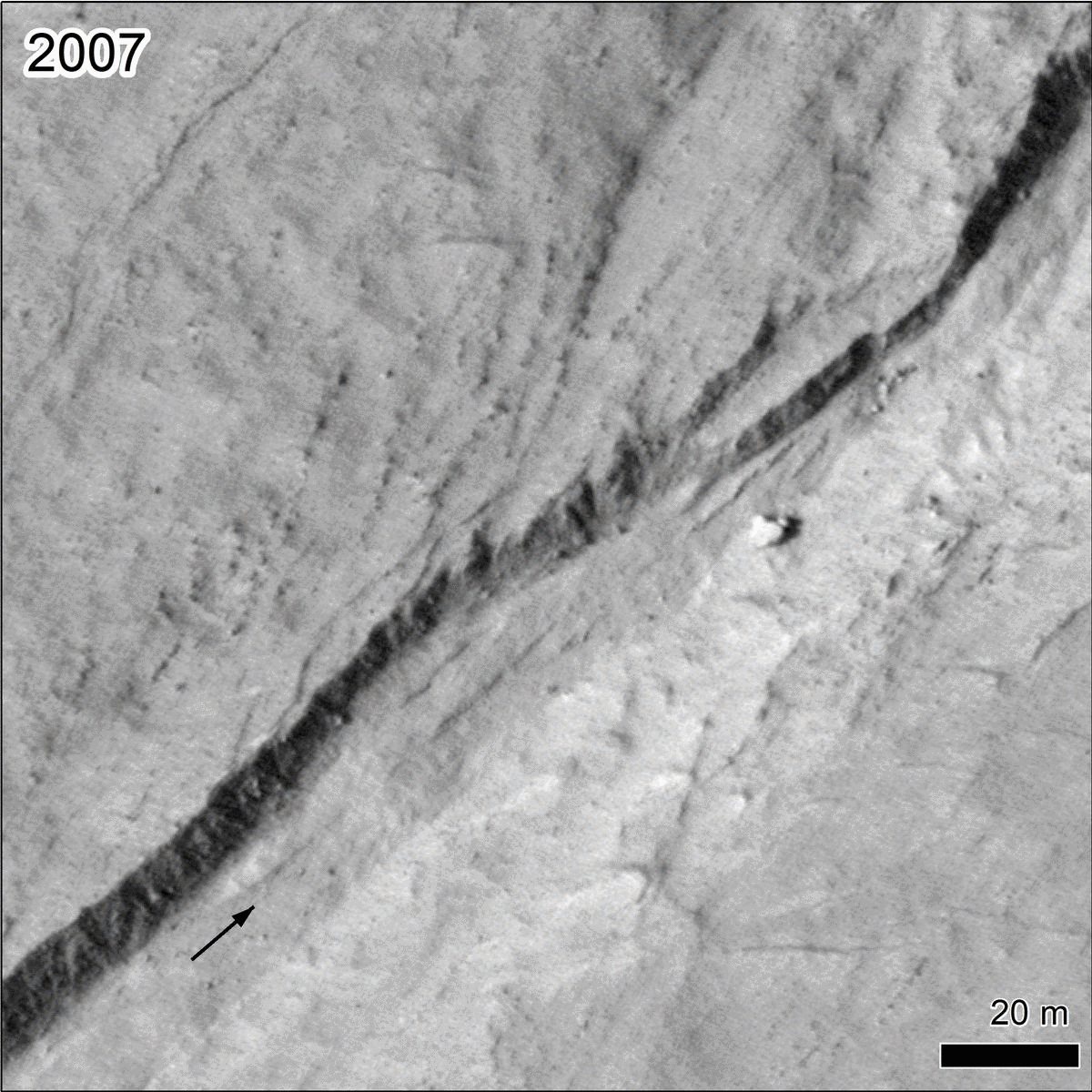

Supplement: Supplementary file 9 — Movie S8 [file JGRE-124-2246-s010.gif]

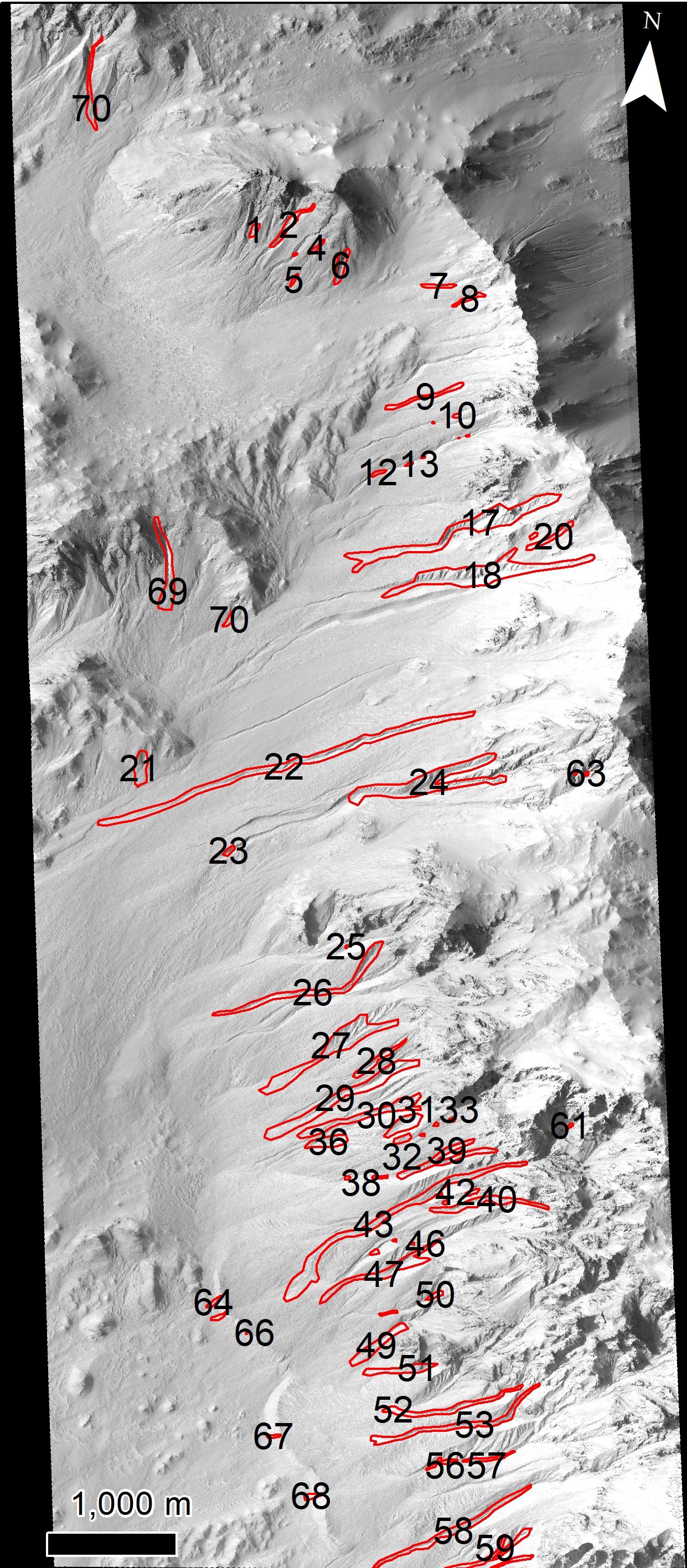

Supplement: Supplementary file 10 — Figure S1 [file JGRE-124-2246-s001.jpg]

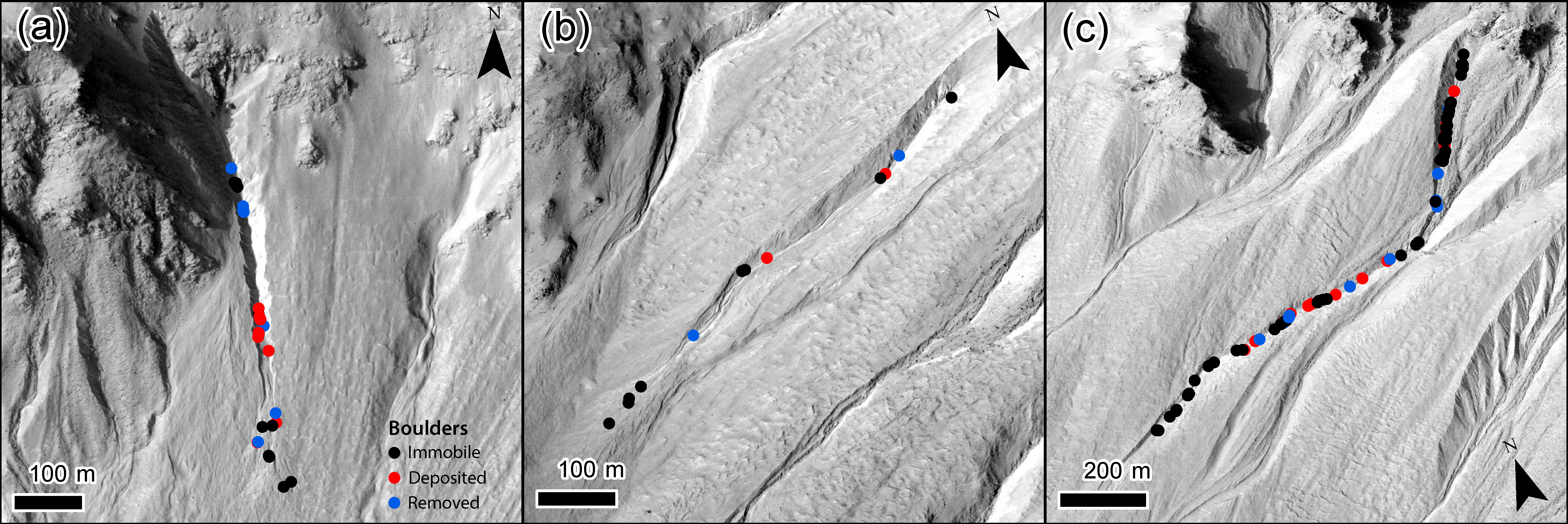

Supplement: Supplementary file 11 — Figure S2 [file JGRE-124-2246-s002.jpg]
